# Inhibition of the Prokaryotic Pentameric Ligand-Gated Ion Channel ELIC by Divalent Cations

**DOI:** 10.1371/journal.pbio.1001429

**Published:** 2012-11-20

**Authors:** Iwan Zimmermann, Alessandro Marabelli, Carlo Bertozzi, Lucia G. Sivilotti, Raimund Dutzler

**Affiliations:** 1 Department of Biochemistry, University of Zürich, Zürich, Switzerland; 2 Department of Neuroscience, Physiology and Pharmacology, University College London, London, United Kingdom; Harvard Medical School, United States of America

## Abstract

The modulation of pentameric ligand-gated ion channels (pLGICs) by divalent cations is believed to play an important role in their regulation in a physiological context. Ions such as calcium or zinc influence the activity of pLGIC neurotransmitter receptors by binding to their extracellular domain and either potentiate or inhibit channel activation. Here we have investigated by electrophysiology and X-ray crystallography the effect of divalent ions on ELIC, a close prokaryotic pLGIC homologue of known structure. We found that divalent cations inhibit the activation of ELIC by the agonist cysteamine, reducing both its potency and, at higher concentrations, its maximum response. Crystal structures of the channel in complex with barium reveal the presence of several distinct binding sites. By mutagenesis we confirmed that the site responsible for divalent inhibition is located at the outer rim of the extracellular domain, at the interface between adjacent subunits but at some distance from the agonist binding region. Here, divalent cations interact with the protein via carboxylate side-chains, and the site is similar in structure to calcium binding sites described in other proteins. There is evidence that other pLGICs may be regulated by divalent ions binding to a similar region, even though the interacting residues are not conserved within the family. Our study provides structural and functional insight into the allosteric regulation of ELIC and is of potential relevance for the entire family.

## Abstract 2

The prokaryotic pentameric ligand-gated ion channel ELIC is inhibited by divalent cations, which occupy a specific extracellular site and interfere with channel gating.

## Author Summary

Pentameric ligand-gated ion channels (pLGICs) are ionotropic neurotransmitter receptors that mediate electrical signaling at chemical synapses. The pLGIC family includes receptors for acetylcholine, serotonin, GABA and glycine, which share a similar structural organization and activation mechanism: the channels are closed in the absence of ligands and open when neurotransmitters bind to a conserved site in the extracellular domain. In many family members, activation by the neurotransmitter can be affected by modulators (including several drugs in therapeutic use), which bind to different sites on the channel. Channel function can be modulated also by divalent cations, which either potentiate or inhibit pLGICs at physiological concentrations. Here, we analyze this mechanism in the pLGIC ELIC, a prokaryotic family member of known structure. We show that divalent cations such as calcium or zinc inhibit ELIC by occupying an extracellular site remote from the ligand-binding region thereby interfering with gating. Although the site of interaction is not conserved between different family members, we present evidence that regulation of other pLGICs involves the same region. Our study has thus provided insights into a regulatory process that appears to be general for the pLGIC family in both eukaryotes and prokaryotes.

## Introduction

The pentameric ligand-gated ion channels (pLGICs) are ionotropic neurotransmitter receptors, which are activated by the binding of ligands to specific sites of the protein. The family includes both cation-selective channels, such as nicotinic Acetylcholine- (nAChRs) and Serotonin receptors (5HT_3_Rs), and anion-selective channels, such as GABA- (GABARs) and Glycine receptors (GlyRs) [1]. Despite these differences in ion selectivity, the overall molecular architecture and the mechanism by which ligands open the ion conduction path are conserved [2]–[8]. pLGIC subunits form either homo- or hetero-pentamers that consist of at least two functional units, an extracellular ligand-binding region and a transmembrane pore [9],[10]. Agonists open the channel by binding to a conserved site in the extracellular domain, at the interface between two subunits [11],[12]. A homomeric receptor contains five equivalent agonist binding sites, several of which need to be occupied for maximum channel activation and this makes the process highly cooperative [5],[13]–[16]. Agonist binding is accompanied by conformational rearrangements that are transmitted over a distance of tens of angstroms from the extracellular domain, via the domain interface to the pore [17]. These receptors have thus become important model systems for the study of allosteric mechanisms [18]. Many pLGICs are important drug targets and all aspects of their function can be influenced by pharmacological agents. These are a diverse set of molecules that include agonists and competitive antagonists (which act on the agonist binding site itself), pore blockers that inhibit ion conduction, and allosteric modulators that interact with regions distinct from the agonist-binding site. Modulators such as benzodiazepines [19], general anesthetics [20], alcohol [21], and the antiparasite ivermectin [22] can either enhance or inhibit pLGIC activation. pLGIC function is affected also by divalent cations (such as calcium and zinc) in two distinct ways. Cation-selective pLGICs are somewhat permeable to divalents, but the strong interaction between these ions and the pore decreases or blocks conduction in a voltage-dependent manner [23],[24]. In addition to that, divalent cations can also modulate channel gating. For instance, calcium potentiates the agonist responses of nAChRs [25]–[27] and inhibits those of 5HT_3_Rs [28],[29], and zinc can either potentiate or inhibit channel activation, depending on the type of pLGIC and the ion concentration [30]–[35].

Here we show that both the modulatory and the channel block effects of divalent cations are present also in ELIC, a prokaryotic pLGIC channel whose structure was determined in a nonconducting conformation [36]. Agonists of ELIC include primary amines such as cysteamine, propylamine, and the vertebrate neurotransmitter GABA. In ELIC, these agonists occupy the canonical ligand-binding site of the family and open a cation-selective pore with permeation properties similar to those of eukaryotic channels [37]. Here we describe how divalent cations permeate and block the ELIC pore, and how they also inhibit ELIC gating, by binding in the extracellular domain, to a site remote from the ligand-binding region.

## Results

### Modulation of ELIC Function by Divalent Ions

**Figure 1:**
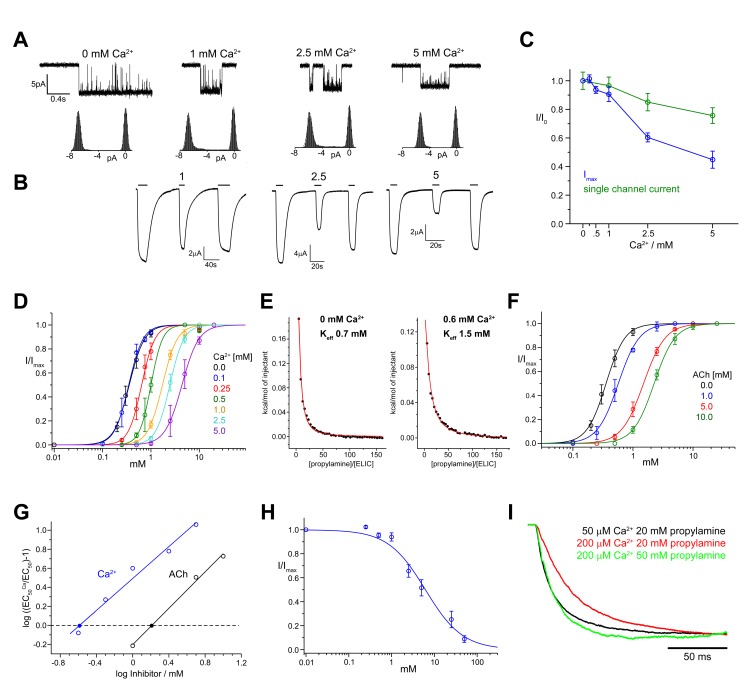
Inhibition of ELIC by calcium. (A) ELIC single channel currents in the presence of different extracellular concentrations of Ca^2+^ and all-points amplitude histograms (recordings were from oocyte outside-out patches at −80 mV holding potential). (B) Maximum ELIC responses to saturating concentrations of the agonist cysteamine in the absence and presence of Ca^2+^. Currents were measured from oocytes held at −40 mV under two-electrode voltage clamp. Agonist application is indicated by a bar. Responses to cysteamine in the absence of extracellular Ca^2+^ are followed by responses in the presence of Ca^2+^ (concentration in mM as shown) and by recovery application of cysteamine in the absence of Ca^2+^. (C) Plot of maximum agonist responses and single channel currents at different Ca^2+^ concentrations. The currents are normalized to the control values (in the absence of Ca^2+^). Maximum cysteamine currents (blue symbols) were measured with the two-electrode voltage clamp technique in oocytes as in panel B. Single channel currents (green symbols) were measured in the outside-out configuration as in panel A. (D) Cysteamine dose–response relationships for ELIC in the presence of different concentrations of Ca^2+^. (E) Equilibrium cysteamine binding isotherms determined by ITC for ELIC in the absence (left) and presence of Ca^2+^. Solid curves represent fits to a single-site binding isotherm with Keff = 0.7 mM (no Ca^2+^) and 1.5 mM (0.6 mM Ca^2+^), respectively. (F) Cysteamine dose–response relationships for ELIC in the presence of different concentrations of ACh. (G) Schild plot quantifying the inhibition by Ca^2+^ and ACh. EC_50_ values were obtained from fits to data shown in panels (D) and (F). Potencies of the antagonists (pA values) were obtained by linear regression, and the intersection with the *x*-axis is indicated (•). (H) Fraction of the maximum current response at different Ca^2+^ concentrations. The solid line shows a fit to a Langmuir equation with a K_i_ of 6 mM. The data presented in panels (C), (D), (F), and (H) are averages from at least five oocytes; errors are SD. The solid lines in (D) and (F) show fits to a Hill equation. Currents were recorded at −40 mV. (I) Activation kinetics of macroscopic currents of ELIC activated by propylamine in response to fast solution exchange at different Ca^2+^ concentrations. ELIC was expressed in HEK 293 cells, and currents were recorded from excised patches in the outside-out configuration at −100 mV.

We have investigated the effects of divalent cations on ELIC by electrophysiology and X-ray crystallography. Divalent cations can influence ELIC function in several different ways depending on concentration (Figure 1). The traces in Figure 1A show that low mM concentrations of the alkaline earth metal ion Ca^2+^ decrease the single channel conductance of ELIC when added to the extracellular medium at negative holding potentials. ELIC single channel currents are progressively reduced by increasing Ca^2+^ concentrations and decrease by approximately 25% of their control amplitude at 5 mM Ca^2+^ (Figure 1C) and by a maximum of about 50% at high Ca^2+^ concentration [37]. This effect is due to tight interactions of divalent ions with the channel pore and has been thoroughly characterized for different pLGIC family members [23],[24] including the homologous channel GLIC [38], whose structure was determined by X-ray crystallography in a conducting conformation [39],[40].

Low extracellular calcium (greater than 100 µM) produces also a voltage-independent decrease in agonist potency. This effect is detectable at Ca^2+^ concentrations too low to decrease channel conductance and is manifested as a parallel rightward shift in the agonist dose–response curve (Figure 1D, Table S1). A similar effect on agonist binding in the presence of calcium is observed in isothermal titration calorimetry experiments (Figure 1E). Up to 1 mM calcium, the shift in the agonist dose–response curves is truly parallel, as the maximum agonist current does not decrease more than the single channel conductance does (Figure 1B and 1C). This pattern appears to reproduce the effects of competitive antagonists, which bind to the ligand-binding site and reduce its occupancy by the agonist in a surmountable way (e.g., their effect can be overcome by increasing agonist concentration). This resemblance is obvious if the effects of Ca^2+^ are compared with those of the competitive antagonist acetylcholine, which is known to bind to the agonist-binding site of ELIC (Figure 1F) [41]. The Schild plot for acetylcholine [42],[43] is linear with a slope of unity and a binding affinity of 1.6 mM (Figure 1G, Table 1). The Schild plot for Ca^2+^ is also linear, with a potency of 260 µM, but a shallower slope of 0.8 (Figure 1G, Table 1).

**Table 1:** Schild analysis of inhibition of ELIC.

ELIC	Inhibitor	pA	Slope	K_i_^app^ [mM]
WT	ACh	0.21±0.06	1.0±0.1	1.6
WT	Ca^2+^	−0.57±0.03	0.8±0.05	0.26
WT/BAPTA	Ca^2+^	−0.69±0.11	1.0±0.3	0.20
WT	Mg^2+^	−0.36±0.02	0.9±0.01	0.43
WT	Sr^2+^	−0.07±0.02	0.9±0.02	0.85
WT	Ba^2+^	−0.07±0.01	1.1±0.03	0.85
WT	Zn^2+^	−2.16±0.13	1.2±0.01	0.007
R91A	Ca^2+^	−0.69±0.03	0.7±0.1	0.21
R91A	ACh	−0.77±0.05	0.7±0.1	0.17
R91A	TMA	0.78±0.18	0.8±0.1	6.0
D86A	Ca^2+^	−0.60±0.11	0.8±0.1	0.25
S84A	Ca^2+^	−0.83±0.14	0.6±0.2	0.15
N251A	Ca^2+^	−0.27±0.19	0.9±0.2	0.53
D113A	Ca^2+^	0.69±0.04	1.0±0.3	4.9
D113N	Ca^2+^	−0.02±0.08	1.0±0.01	0.95
D158A	Ca^2+^	0.74±0.01	1.2±0.1	5.4
D158N	Ca^2+^	0.50±0.17	0.5±0.2	3.2
E150A	Ca^2+^	0.23±0.12	1.0±0.2	1.7
E150Q	Ca^2+^	0.09±0.20	0.9±0.3	1.2
D113A/D158A	Ca^2+^	N/D	N/D	N/D
D113A/D158A	Ba^2+^	0.81±0.02	0.6±0.02	6.5
D113A/D158A	Zn^2+^	N/D	N/D	N/D
D113A/D158A	Ach	0.32±0.06	0.8±0.1	2.1
WT/.25 mM Ca^2+^	Ach	0.06±0.06	0.9±0.1	1.2
WT/.5 mM Ca^2+^	Ach	0.11±0.09	0.9±0.01	1.3
WT/1 mM Ca^2+^	Ach	0.35±0.01	1.1±0.01	2.2
WT/1 mM ACh	Ca^2+^	−0.65±0.01	0.9±0.02	0.22

The similarity between the effect of calcium and that of a competitive antagonist disappears as Ca^2+^ concentrations are increased above 1 mM. The current traces in Figure 1B show that the reduction in agonist potency is now associated with a decrease in the maximum agonist response. This decrease is too big to be explained by the effect of Ca^2+^ on conductance: at 5 mM Ca^2+^ the single channel conductance is reduced by 25% and the maximum agonist response by 55% (Figure 1C). At progressively higher concentrations of the divalent cation, the maximum current response continues to decline and this decrease can be described by a fit to a Langmuir equation with an IC_50_ of 6 mM (Figure 1H). Despite the strong reduction in the maximum currents, the shift in EC_50_ remains linear over a wide concentration range (Figure S1). The pronounced drop in maximum current strongly suggests that at higher concentrations calcium impairs the opening of the channel and reduces agonist efficacy.

Next, we tried to establish whether calcium impairs the maximum rate of ELIC gating (e.g., when the channel is fully bound to the agonist) by measuring the on-relaxation of currents elicited by rapid propylamine applications to outside-out patches from HEK293 cells. Figure 1I shows that increasing Ca^2+^ from 50 to 200 µM does slow the onset of the current elicited by a saturating agonist concentration (20 mM propylamine, red trace) but that this effect is overcome by increasing agonist concentration to 50 mM (green trace). Only minor changes in the time course of deactivation were detected (Table 2). Thus the maximum rate with which the agonist-bound channel opens is unchanged, which is unexpected given the observed change in agonist efficacy. This could be because we could test only low calcium (in high calcium the concentrations of agonist required to saturate channel gating are too high to be experimentally feasible). Alternatively, calcium impairs gating by affecting a step in the channel activation that controls the size of the maximum agonist response, but not the speed of overall gating (see Discussion).

**Table 2:** Activation and deactivation kinetics of ELIC.

Ca^2+^ (µM)	Propylamine (mM)	τ_rise_ (ms)	τ_decay_ (ms)	*n*
50	20	16.7±1.5	34.7±5.6	6
200	20	38.5±6.7	23.9±3.1	6
200	50	16.4±2.2	36.7±12.7	6
50	20	19.6±3.1	33.5±6.9	6

**Figure 2:**
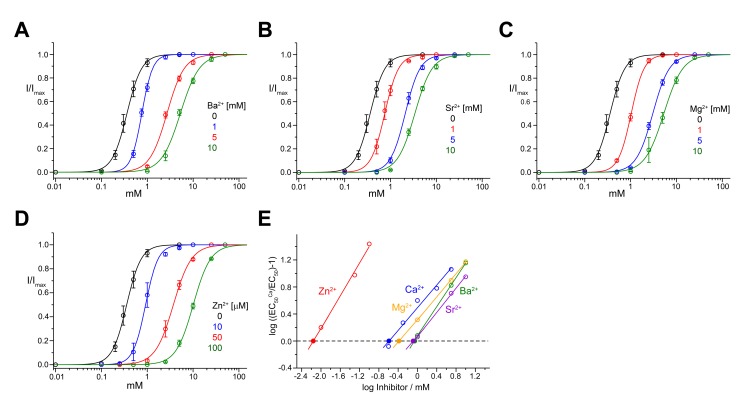
Inhibition of ELIC by different divalent cations. Dose–response relationships of ELIC activated by cysteamine at different concentrations of Ba^2+^ (A), Sr^2+^ (B), and Mg^2+^ (C) and ELIC activated by propylamine at different concentrations of Zn^2+^ (D). (E) Schild plot quantifying the inhibition by different divalent cations. EC_50_ values were obtained from fits to data shown in panels (A–D). Potencies of the Antagonists (pA values) were obtained by linear regression, and the intersection with the x-axis is indicated. The data presented in panels (A–D) are averages from at least 5 oocytes; errors are SD. The solid lines show fits to a Hill equation. Currents were recorded at −40 mV.

Finally, we found that divalent cations other than Ca^2+^ also affect ELIC responses. In particular, other alkaline earth metal ions, such as Mg^2+^, Sr^2+^, and Ba^2+^, are slightly weaker than Ca^2+^ in inhibiting ELIC (Figure 2A–C and E, Table 1, Table S1), whereas the transition metal ion Zn^2+^ is considerably more potent (i.e., Schild plot x-intercept 8 µM, Figure 2D and E, Table 1, Table S1).

### Structural Characterization of Divalent Ion Binding

In order to understand the structural basis of the effects of divalent ions we aimed at identifying the region of interaction by X-ray crystallography. Since the crystal form that was used for the structure determination of ELIC contains high concentrations of sulfate, which forms insoluble salts in the presence of most alkaline earth metal ions, we had to identify novel crystallization conditions compatible with divalent ions. In a broad screen we observed crystals growing in Ba^2+^-acetate. Ba^2+^ can be readily located in the electron density by its strong anomalous scattering properties, and since it has comparable effects on channel function as Ca^2+^ (Figure 2A, Figure S2A), it is reasonable to assume that it will occupy the same sites in the protein. Crystals of the ELIC/Ba^2+^ complex belong to two different, yet related crystal forms, one similar to the original barium-free form of ELIC that was used for structure determination (space group P2_1_) and another growing in a higher symmetry space group (P4_3_) (Table 3). Datasets for both crystal forms were collected to 3.8 Å (P2_1_) and 3.3 Å (P4_3_) resolution and provide equivalent views of the channel and its interaction with divalent cations.

**Table 3:** Data collection and refinement statistics.

Crystallography	WT Ba^2+^	WT Ba^2+^	N251A Ba^2+^	D113A/D158A Ba^2+^	WT TMAs
**Data collection**					
Space group	P4_3_	P2_1_	P2_1_	P2_1_	P2_1_
Cell dimensions					
*a*, *b*, *c* (Å)	100.4, 100.4, 263.7	104.6, 267.5, 109.3	101.5, 268.5, 101.2	103.7, 266.7, 108.8	105.4, 266.8, 110.9
*α*, *β*, *γ* (°)	90, 90, 90	90, 112.6, 90	90, 108.6, 90	90, 112.8, 90	90, 109.6, 90
Resolution (Å)	40-3.3	40-3.8	40-3.7	40-4.4	40-4.0
*R*_merge_	12.5 (64.6)	11.9 (69.9)	11.8 (81.0)	12.6 (84.3)	11.0 (81.8)
*I*/σ*I*	12.1 (2.7)	7.6 (2.1)	8.1 (2.1)	8.6 (2.5)	8.0 (1.5)
Completeness (%)	99.4 (96.6)	99.3 (99.0)	96.7 (96.2)	98.6 (97.7)	97.1 (85.6)
Redundancy	6.5 (6.1)	3.4 (3.5)	3.5 (3.6)	4.7 (4.7)	3.1 (3.0)
**Refinement**					
Resolution (Å)	20-3.3	30-3.8	30-3.7	30-4.4	30-4.0
*R*_work/_ *R*_free_	22.7/25.8	25.5/27.2	24.5/27.4	21.6/23.9	25.6/27.0
Wilson B-factor	90	86	109	107	102
R.m.s. deviations					
Bond lengths (Å)	0.01	0.01	0.01	0.01	0.01
Bond angles (°)	1.2	1.2	1.0	1.3	1.2

Values in parentheses are for highest resolution shell. R.m.s., root mean square.

**Figure 3:**
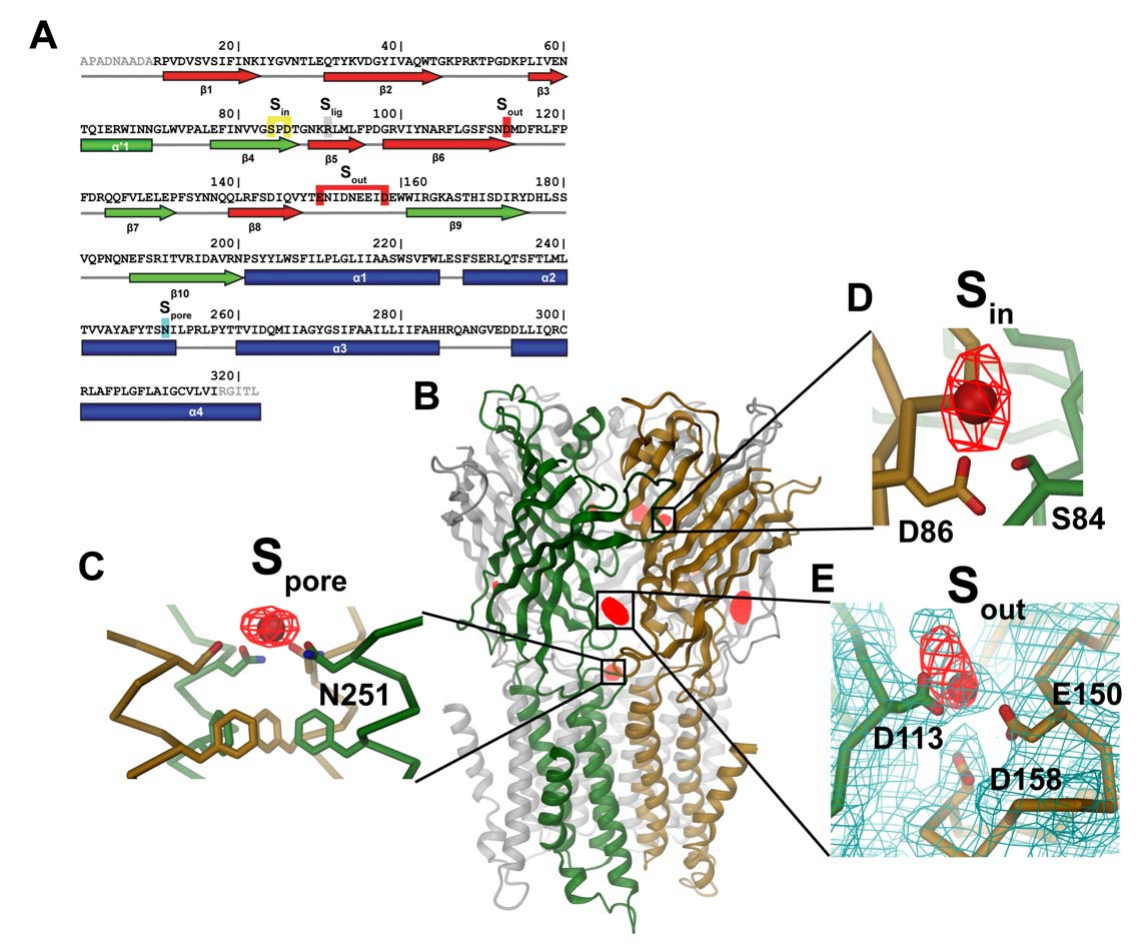
Structure of ELIC in complex with divalent cations. (A) Sequence of ELIC with secondary structure elements indicated below. Residues contributing to ion coordination in different sites are highlighted (S_in_, yellow; S_out_, red; S_pore_, cyan; Arg 91 in the ligand-binding pocket, S_lig_, grey). (B) Anomalous difference electron density of ELIC in complex with Ba^2+^ superimposed on the structure of the ELIC pentamer (shown as ribbon representation). Ion-binding sites are labeled. Close-up of S_pore_ (C) and S_in_ (D). The protein is shown as Cα-trace with selected side-chains close to Ba^2+^ (red sphere) shown as sticks. (E) Close-up of S_out_. The protein is shown as Cα-trace with selected side-chains close to Ba^2+^ (red sphere) shown as sticks. The 2F_o_-F_c_ electron density of a dataset from a crystal of space group P2_1_ was calculated at 3.8 Å and contoured at 1 σ (shown in cyan). The refined model used to calculate phases did not contain Ba^2+^-ions. The anomalous difference electron densities shown in (B–E) (red mesh) were calculated from the same dataset at 5 Å and contoured at 5 σ. Crystals of space group P4_3_ showed a qualitatively similar picture. Structures in Figures 3–7 were prepared with DINO (www.dino3d.org).

The structures show a conformation of the channel that is overall very similar to the structure of ELIC already described. Strong peaks in the anomalous difference density allow us to detect the presence of Ba^2+^ ions bound to three distinct sites of the protein (Figure 3).

Firstly, a single Ba^2+^ ion per channel is located on the 5-fold axis of symmetry at the extracellular end of the pore and is coordinated by the side-chains of Asn251 (position 20′ of the second transmembrane domain in the numbering system developed for the nAChR, Figure 3A–C). Throughout the article we will refer to this site as S_pore_.

There are two additional sets of binding sites for Ba^2+^ in the structure shown in Figure 3B. Both are found at the interface between subunits in the extracellular domain in five symmetry-related locations. One set of sites faces the channel vestibule and will be referred to as S_in_. The barium ion in S_in_ is coordinated by Ser84 of the principal subunit and Asp86 of the complementary subunit (Figure 3D). Barium ions are bound also to a set of five equivalent sites on the outer rim of the extracellular domain (Figure 3B and E). These sites, which we will call S_out_, are about 15 Å below the ligand-binding pocket, towards the membrane plane and are formed by the side-chains of acidic amino acids contributed by both subunits. These residues include Asp113 at the end of β6 on the principal side and Glu150 and Asp158 on the loop connecting β8 and β9 on the complementary side (Figure 3A and E). The refined 2F_o_-F_c_ electron density map of this region indicates a direct interaction of the respective carboxylate groups with the bound ions resembling Ca^2+^-binding sites observed in other proteins (Figure 3E, Figure S2B and C). Remarkably, in none of the collected datasets did we find any evidence for Ba^2+^ in the ligand-binding pocket itself.

### Investigation of the Binding Sites of Divalent Ions by Mutagenesis

**Figure 4:**
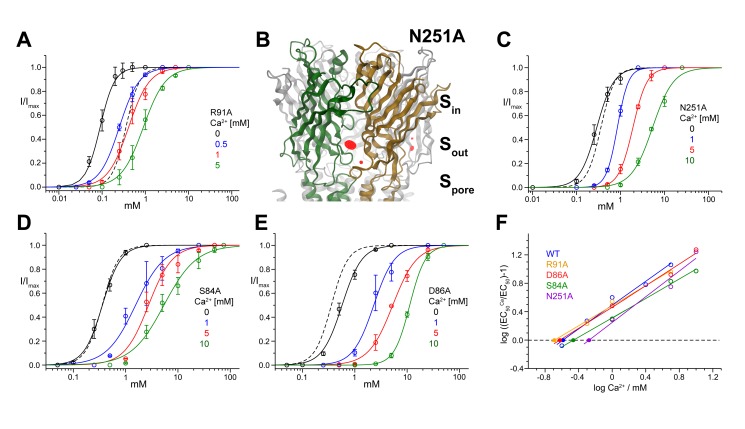
Divalent ion inhibition in mutants of the nonregulatory sites S_pore_ and S_in_. Dose–response relationships of the ELIC point mutant R91A (A) activated by cysteamine at different concentrations of Ca^2+^. (B) Anomalous difference electron density (calculated at 5 Å and contoured at 5 σ) from data of the mutant N251A in complex with Ba^2+^ superimposed on a model of ELIC in ribbon representation. Ion-binding sites are indicated. Dose–response relationships of the ELIC point mutants N251A (C), S84A (D), and D86A (E) activated by cysteamine at different concentrations of Ca^2+^. The data presented in panels (A), (B), (D), and (E) are averages from at least 5 oocytes; errors are SD. The solid lines show fits to a Hill equation. Currents were recorded at −40 mV. A dose–response curve of WT in the absence of Ca^2+^ (dashed line) is shown for comparison. (F) Schild plots quantifying the inhibition of ELIC mutants by Ca^2+^. EC_50_ values were obtained from fits to data shown in panels (A), (B), (D), and (E). Potencies of the antagonists (pA values) were obtained by linear regression; the intersection with the *x*-axis is indicated (•). WT is shown for comparison.

**Figure 5:**
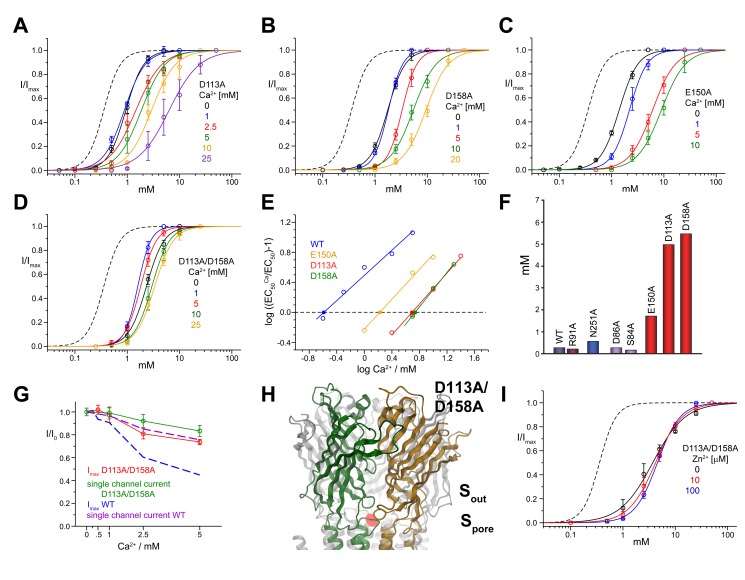
Divalent ion inhibition in mutants of the regulatory site S_out_. Dose–response relationships of the ELIC mutants D113A (A) and D158A (B), E150A (C), and the double mutant D113A/D158A (D) activated by cysteamine at different concentrations of Ca^2+^ are shown. (E) Schild plots quantifying the inhibition of ELIC mutants by Ca^2+^. EC_50_ values were obtained from data shown in panels (A–D). Potencies of the antagonists (pA values) were obtained by linear regression; the intersection with the *x*-axis is indicated (•). WT is shown for comparison. (F) Graphical depiction of potencies for Ca^2+^ inhibition in different mutants. (G) Maximum current response of the double mutant D113A/D158A at different Ca^2+^ concentrations. The currents are normalized to the maximum response in the absence of Ca^2+^. Whole cell currents measured at −40 mV with the two-electrode voltage clamp technique are shown in red (the averages of at least 5 oocytes are shown; errors are SD). Single channel currents from the double mutant D113A/D158A were measured in the outside-out configuration and are shown in green. WT macroscopic and single channel currents are shown as dashed lines for comparison. (H) Anomalous difference electron density (calculated at 5 Å and contoured at 5 σ) from data of the double mutant D113A/D158A in complex with Ba^2+^ is superimposed on a model of ELIC in ribbon representation. Ion-binding sites are indicated. (I) Dose–response relationships of the double mutant D113A/D158A activated by propylamine at different concentrations of Zn^2+^. The data presented in panels (A–D) and (I) are averages from at least 5 oocytes; errors are SD. The solid lines show fits to a Hill equation. Currents were recorded at −40 mV. A dose–response curve of WT in the absence of Ca^2+^ (dashed line) is shown for comparison.

The structure of ELIC in complex with Ba^2+^ has revealed the location of three distinct sites for the interaction with divalent cations. If binding to any of these sites is relevant for the inhibition of the channel, we would expect that mutating the interacting residues should affect the functional modulation by divalent ions. Thus we mutated the residues that contact Ba^2+^ in the structure and measured again the effects of Ca^2+^ by two-electrode voltage-clamp electrophysiology (Figures 4 and 5, Table 1).

Given that the effects of low Ca^2+^ concentrations resemble those of competitive antagonists, we tested also whether the agonist binding site can play a role (even though we have no structural evidence that divalents bind there). Our functional data show that the agonist binding site is unlikely to be involved, because Ca^2+^ inhibition is not changed by a mutation here (R91A) that increases agonist potency by 3–4-fold ([37], Figure 4A and F).

We then proceeded to investigate whether the inhibitory effects of Ca^2+^ are produced via binding to the S_pore_ site by truncating the side-chain of the Asn residue in contact with the divalent ion. Our X-ray crystallography data show that the structure of this N251A mutant is on the whole similar to WT but lacks the anomalous difference density in S_pore_. The structure of this mutant still shows strong density of ions bound to S_out_ (and weaker density for S_in_), thus suggesting that effects of the mutation are local (Figure 4B). Electrophysiological recording shows that agonists activate WT and mutant N251A channels with similar potency and that the inhibition by Ca^2+^ of these responses is only modestly decreased in N251A (Figure 4C and Schild plots in 4F). This suggests that S_pore_ is not the major site responsible for the Ca^2+^ inhibition.

Figure 4F shows also that mutating the binding residues in another set of divalent ion sites, S_in_ (which face the extracellular vestibule), has little effect on Ca^2+^ inhibition. Mutation S84A (on the principal side) changes neither the potency of the agonist nor the inhibition by Ca^2+^ (Figure 4D). Similarly, in the mutant D86A there is only a modest decrease in agonist potency, and the inhibitory effect of Ca^2+^ is virtually unchanged (Figure 4E and F). Thus we have shown that neither S_pore_ nor S_in_ mediate the functional effects of calcium on channel activation.

In contrast to that, we found that Ca^2+^ modulation is greatly decreased when we change any of the residues that coordinate divalent cations in S_out_. This is seen both when the residues with acidic side chains (Asp 113, Glu150, and Asp158) are individually replaced with their uncharged isosteric counterparts (Asn or Gln) and when the acidic side-chains are truncated to Ala (Figure 5, Figure S3). All of these mutations cause a variable but strong decrease in the potency of Ca^2+^, which suggests that they weaken the interaction with the ion and thus its inhibitory effects (Figure 5E and F). The strongest effect among single mutants is observed for residues Asp113 and Asp158 (Figure 5A, B, and E). Combining these two mutations in the double mutant D113A/D158A virtually abolishes the effects of both calcium and barium on the agonist dose–response curves (Figure 5D, Figure S3E and S3F). Remarkably, and in contrast to our observations in WT, in this double mutant the decrease in I_max_ at high Ca^2+^ concentration appears entirely due to the reduction in single channel conductance (Figure 5G). The binding of Ca^2+^ to S_out_ is thus responsible for both functional effects on the shift of the EC_50_ and the decrease of I_max_. Figure 5 also shows that mutations in S_out_ shift the EC_50_ towards higher agonist concentrations, an effect that is not surprising given that this region is thought to be important in transducing agonist binding into channel activation (Figure 5A–D, Figure S3, Table S1).

The X-ray structure of the double mutant D113A/D158A in complex with Ba^2+^ is on the whole unperturbed. The double mutation has removed the density of ions bound to S_out_, while leaving the strong anomalous difference density in S_pore_ unchanged. This confirms that in this mutant divalents fail to modulate channel activation because they cannot bind to the S_out_ site (Figure 5H).

Given that the same mutations abolish also the modulation by Zn^2+^ (Figure 5I), it is very likely that Zn^2+^ inhibits ELIC by binding to the same site. This finding is somewhat unexpected as Zn^2+^ usually interacts with histidine or cysteine residues. However, since the ligand binding domain of ELIC does not contain any cysteines and since mutations of the two histidines, which are both located on β10, did not affect the inhibition by Zn^2+^ (Figure S4), it is likely that the interaction of this transition metal ion with ELIC occurs at this site and therefore deviates from common binding modes.

### Independence of Ca^2+^ and Acetylcholine Inhibition

**Figure 6:**
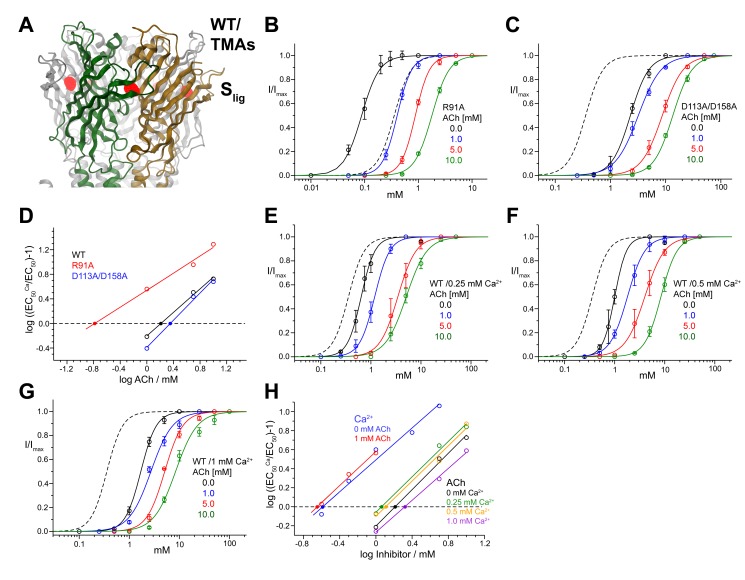
Inhibition by ACh. (A) Anomalous difference electron density (calculated at 5 Å and contoured at 6 σ) from data of WT in complex with TMAs is superimposed on a model of ELIC in ribbon representation. The ligand-binding site is indicated. Dose–response relationships upon activation with cysteamine of the ELIC mutants R91A (B) and the double mutant D113A/D158A (C) at different concentrations of ACh. (F) Schild plots quantifying the inhibition of ELIC mutants by ACh. EC_50_ values were obtained from data shown in panels (B–C). Dose–response relationships upon activation with cysteamine of WT in the presence of either 0.25 (E), 0.5 (F), or 1 mM Ca^2+^ (G). (H) Schild plots quantifying the inhibition of ELIC mutants by ACh in the presence of Ca^2+^. EC_50_ values were obtained from data shown in panels (E–G). Potencies of the antagonists (pA values) were obtained by linear regression; the intersection with the *x*-axis is indicated (•). The data presented in panels (B–C) and (E–G) are averages from at least 5 oocytes; errors are SD. The solid lines show fits to a Hill equation. Currents were recorded at −40 mV. A dose–response curve of WT in the absence of ACh and Ca^2+^ (dashed line) is shown for comparison.

The results of our mutational analysis strongly suggest that the observed inhibition of ELIC by divalent cations is mediated by the specific interaction with a site that is located at the outer rim of the extracellular domain, at the interface between neighboring subunits. Since this site is distant from the agonist-binding region, we wanted to explore whether there is any direct competition between the effect of divalent ions and that of competitive antagonists binding to the ligand-binding site. Such competitive antagonists include quaternary ammonium compounds such as tetramethylammonium, a weak antagonist (Figure S5), or acetylcholine, which inhibits the channel with higher affinity. The X-ray structure of ELIC in complex with the heavy atom analogue tetramethylarsonium (Figure 6A) and the recently determined structure of ELIC in complex with acetylcholine [41] show that both antagonists bind to the ligand-binding pocket and prevent the binding of the agonist to the same site. The overlap of agonist- and antagonist-binding sites is also reflected in the 10-fold increase in the Schild affinity of acetylcholine in the mutant R91A. This is similar to the increase in agonist potency in the same mutant (Figure 6B and D). In contrast to the mutation in the binding site, the S_out_ double mutant D113A/D158A abolishes the modulatory effect of Ca^2+^ but does not alter the affinity of acetylcholine (WT 1.6 mM, D113A/D158A 2.1 mM), confirming that calcium and acetylcholine act via distinct sites (Figure 6C and F, Table 1).

Finally, in order to probe whether the presence of one antagonist would alter the effect of the other, we have studied the inhibition of ELIC by acetylcholine in the presence of different concentrations of Ca^2+^ and vice versa. In no case did we find any significant change in the potency of either antagonist, which suggests that the inhibitory effects are additive and the two compounds thus act independently (Figure 6E–H).

## Discussion

In the present study we have investigated how divalent cations modulate the function of ELIC, a bacterial member of the pLGIC family. ELIC is inhibited by alkaline earth metal ions and by the transition metal ion zinc. The modulation reported here resembles similar effects observed in other family members, where divalent cations act as either positive or negative modulators of gating. Ca^2+^ potentiates channel activity in a subset of nAChRs [26],[27], whereas it has an inhibitory effect on 5HT_3_Rs [29]. Like in ELIC, in 5HT_3_Rs calcium shifts the EC_50_ of activation towards higher ligand concentrations [29]. The action of Zn^2+^ appears to be more complex. In some subtypes of GABARs, Zn^2+^ inhibits channel activity [31], whereas in GlyRs, nAChRs, and 5HT_3_Rs, it acts as a potentiator at low concentrations and as an inhibitor at higher concentrations [30],[32],[33]. These opposing effects are believed to be mediated by the successive occupation of binding sites of different affinity.

### Divalent Ions Inhibit ELIC Gating by Binding to S_out_

**Figure 7:**
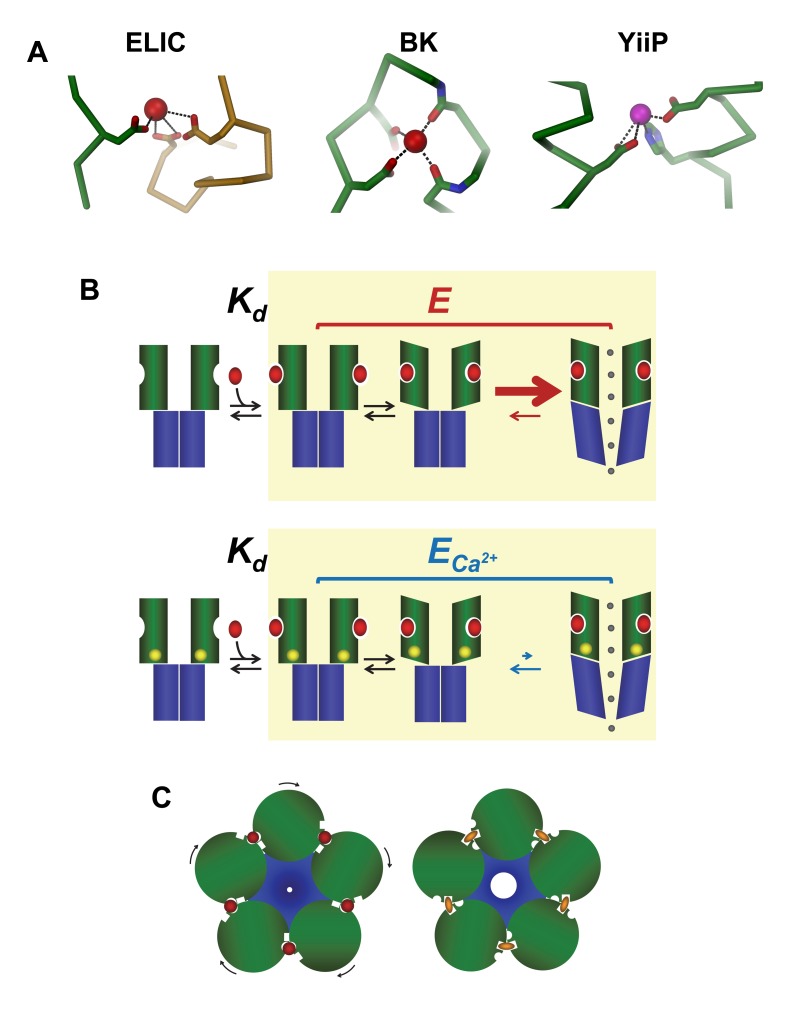
Potential mechanisms. (A) Interactions in the regulatory divalent ion-binding site of ELIC (left) in comparison with a regulatory Ca^2+^ binding site of the BK-channel (middle) and the Zn^2+^-transporter YiiP (right). (B) Schematic model of a potential mechanism for the inhibitory effect of divalent ions. The two rows show simplified schemes for channel activation in control conditions (top) and in the presence of divalent ions. From left to right, the schemes show that binding of agonist molecules (red ovals) to the extracellular domain (with microscopic affinity *K*_d_) is followed by conformational changes (yellow background) that result in channel opening. Channel gating (described by the efficacy equilibrium constant *E*) is impaired when the channel is bound to divalent ions (yellow circles, *E*_Ca_^2+^). The decrease in agonist efficacy is likely to be due to a change in the rate of opening, as shown by the size of the arrows in the last step of the reaction. (C) Schematic mechanism of how binding sites located on similar places of an oligomeric channel could alternately stabilize the closed or open conformation of the channel.

X-ray structures of ELIC crystals grown in the presence of barium have allowed us to identify five structurally equivalent binding sites (S_out_) located at subunit interfaces on the extracellular domain about 15 Å from the agonist-binding region. These are likely to be responsible for the observed inhibition, as mutations at this site have a strong effect on the potency of both Ca^2+^ and Zn^2+^. The sites resemble regulatory calcium-binding pockets found in other ion channel proteins, where the divalent ions interact with the side chains of acidic residues that are often organized in clusters on the protein sequence (Figure 7A) [44]–[46]. The interaction found in ELIC is, however, not typical for zinc-binding sites, as these usually contain either histidines or cysteines for ion coordination [47]–[49], residues that are unlikely to play this role in ELIC (Figure 7A, Figure S4).

While the residues that interact with divalent cations in ELIC are not conserved across pLGICs, there is evidence that equivalent modulatory effects in other pLGICs involve the same (S_out_) region. In the α7-nAChR, the residue Glu 172, which has been identified as a key residue in the interaction with calcium [50]–[52], resides on the same loop as Glu 150 and Asp 158 (loop 8) in ELIC. Similarly, histidine and glutamate residues contributing to the interaction with Zn^2+^ in GABA_A_Rs were mapped to the same location, at the interface between two subunits [53], thus indicating that the Zn^2+^-dependent inhibition of GABA_A_Rs may follow a similar mechanism. Residues in the same loop of 5HT_3_Rs have also been proposed to participate to calcium regulation of this receptor [54].

Interestingly, a study on the 5HT_3_R has identified an aspartate residue in the pore domain as an important determinant for calcium-dependent inhibition. The equivalent Asn residue in ELIC coordinates the barium ion in the site S_pore_ [55]. We investigated this site by mutagenesis but did not find any indication for a similar role in the calcium regulation of ELIC. The phenotypic difference may be due to a stronger interaction with a divalent ion in the 5HT_3_R where the respective residue is an aspartate and thus carries a negative charge (cf., an uncharged asparagine in ELIC).

### The Mechanism of Action of Divalent Ions

The effect of calcium and other divalent cations on gating of ELIC results in a complex functional phenotype. At low extracellular calcium concentrations, we see a reduction in agonist potency that resembles competitive inhibition (with a linear Schild plot with a slope near unity). Despite this resemblance, the agonist binding site is not involved in this process and the presence of the antagonist acetylcholine (which binds in the canonical agonist site) has no effect on the action of calcium. Finally, higher calcium concentrations reduce the maximum agonist response (to a greater extent than can be accounted for by a conductance decrease). At first sight, these effects appear to be too complex to be explained by a single microscopic action of divalents (i.e., the binding of Ca^2+^ to the site S_out_). However, they can all be accounted for, if calcium impairs a single step of ELIC activation, for example channel opening, provided gating is efficient in wild-type ELIC (i.e., the agonist efficacy *E* is high to start with, Figure 7B). This is a plausible hypothesis, given the high open probability of the single channel activity in Figure 1A.

In first approximation, the relation between maximum open probability *P*_max_ and efficacy *E* is:
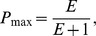
and our observations of an ELIC *P*_max_ greater than 95% are compatible with values of *E* that are greater than 20 (as reported for other pLGIC such as nicotinic and glycine receptors). If the value of *E* is high to start with, the reduction in efficacy produced by divalents must be substantial before a decrease in maximum response becomes apparent. That is why it is seen only at high calcium concentrations. More modest decreases in efficacy, at low calcium concentrations, will cause only a decrease in agonist potency. This is because agonist *EC*_50_ is directly affected by the value of *E*. In the simplest del Castillo-Katz model, *EC*_50_ is given by:
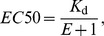
where *K*_d_ is the microscopic dissociation constant of the agonist (Figure 7B) [56].

It can also be shown (Text S1) that the effects of calcium and those of a competitive antagonist are expected to be independent, if we model equilibrium channel activation with a simple scheme, where calcium binding impairs gating (by affecting *E*) and the antagonist binds to the resting form of the channel. This model not only predicts Schild-like behavior for the effect of calcium but suggests also that the Schild intercept is a reasonable estimate for the microscopic affinity of divalents (Text S1).

These conclusions are unchanged if we model channel activation by a more detailed and realistic activation scheme, incorporating an intermediate state between agonist binding and channel opening. The existence of one or more gating intermediate states for channels in the nicotinic superfamily is supported by several lines of evidence. For instance, φ analysis in muscle nicotinic AChRs [57] indicates that blocks of residues move asynchronously in the gating conformational change. In addition to that, mechanisms with reaction intermediates (referred to as flip, primed, or catch-and-hold [13],[58]–[61]) are needed to explain several aspects of the function of the GlyR and the muscle nicotinic AChR, such as agonist efficacy (Figure 7B). In our experiments, the presence of an additional intermediate step that limits the maximum rate of current onset in agonist-bound ELIC channels is required to explain the results of our agonist concentration jumps. This is because we observed that low calcium increased the agonist concentration needed to achieve the maximum rate of current onset, but did not change the limiting rate of channel gating. If activation went through a single conformational step as the channel gates (as in a simple del Castillo-Katz mechanism), this single step would control both the rate of current onset for the agonist-bound channel and the maximum response, and any changes in this would be experimentally detectable (see Text S1).

### Conclusions

In our study we have shown how the binding of calcium to a single site remote from the ligand binding pocket modulates the activation of the pLGIC ELIC. Given that divalent ions impair ELIC gating, they are expected to bind more tightly to the resting state of the channel and stabilize it. The location of the divalent binding site at the interface between adjacent subunits is an intriguing mechanism to stabilize distinct states in an allosteric protein, given that these regions are involved in conformational changes (Figure 7C). Thus, occupancy by divalent ions of sites at a similar location in the different pLGICs will result in potentiation or inhibition, depending on whether the equilibrium is shifted towards conducting or nonconducting conformations. Allosteric modulation is important for the pharmacology of pLGICs, as many of pLGIC drugs in therapeutic use act by this mechanism, although by binding to sites distinct from those of divalent ions.

Modulation by divalent ions of pLGICs occurs at concentrations that are physiologically relevant in vertebrates and may regulate the activity of channels in their natural environment [31],[62]. It is not known whether such regulation is important for ELIC activity in its natural host *Erwinia chrysanthemi*, but it is remarkable that the observed mechanism has been conserved during evolution.

## Materials and Methods

### Protein Expression and Purification

ELIC WT and point mutants were expressed and purified as described [36],[37]. *E. coli* BL21DE3 containing a vector encoding for a fusion protein consisting of the pelB signal sequence, a His_10_ tag, maltose binding protein, a HRV 3C protease site, and ELIC were grown in M9 minimal medium at 37°C to an OD of 1.0 and subsequently cooled to 20°C. Expression was induced by addition of 0.3 mM IPTG and carried out overnight. All the following steps were performed at 4°C. The protein was extracted from isolated membranes in a buffer containing 1% n-Undecyl-β-D-Maltoside (UDM, Anatrace, Inc.) and purified by Ni-NTA chromatography (Qiagen). The purified MBP-ELIC-fusion protein was digested with HRV 3C protease to cleave the His_10_-MBP protein. His_10_-MBP and 3C protease were subsequently removed from solution by binding to Ni-NTA resin. ELIC was concentrated and subjected to gel-filtration on a Superdex 200 column (GE Healthcare). The protein peak corresponding to the ELIC pentamer was pooled and concentrated to 10 mg/ml and used for crystallization.

### Crystallization and Structure Determination

The purified protein was crystallized in sitting drops at 4°C. Protein containing additional 0.5 mg/ml *E. coli* polar lipids (Avanti Polar Lipids, Inc.) was mixed in a 1∶1 ratio with reservoir solution (50 mM ADA pH 6.5, 50 mM BaAc_2_, and 10% (w/v) PEG4000). The crystals were cryoprotected by transfer into solutions containing 30% ethyleneglycol. All datasets were collected on frozen crystals on the X06SA beamline at the Swiss Light Source (SLS) of the Paul Scherrer Institut (PSI) on a PILATUS detector (Dectris). The data were indexed, integrated, and scaled with XDS [63] and further processed with CCP4 programs [64]. The structure of WT and mutants in space groups P4_3_ and P2_1_ were determined by molecular replacement in PHASER [65] using the ELIC pentamer (2VLO) as a search model. G164, which was not included in the original model (2VLO), was introduced according to the structure of the ELIC acetylcholine complex (3RQW). The absence of this amino acid had only a local effect and did not influence the location of neighboring residues. The model was rebuilt in Coot [66] and refined maintaining strong NCS constraints in PHENIX [67]. R and R_free_ were monitored throughout. R_free_ was calculated by selecting 5% of the reflection data in thin slices that were selected for the initial dataset of ELIC and that were omitted in refinement.

### Isothermal Titration Calorimetry

Binding of the agonist propylamine to ELIC in the presence and absence of calcium was measured by isothermal titration calorimetry (ITC) with a MicroCal ITC200 system (GE Healthcare). The syringe was loaded with agonist solution containing 30–37 mM propylamine dissolved in measurement buffer (containing 25 mM Tris-HCl pH8.5, 150 mM NaCl, and in certain experiments 0.6 mM CaCl_2_). The sample cell was loaded with 300 µl of purified ELIC in measurement buffer containing 0.9 mM UDM at a concentration between 80 and 110 µM. Agonist was applied by sequential injections of 2 µl aliquots followed by a 180 s equilibration period after each injection. The data were recorded at 4°C and analyzed by a fit to a single-site binding isotherm.

### Two-Electrode Voltage Clamp Recording

Constructs containing the gene of either the WT or mutant channels preceded by the signal sequence of the chicken α7nAchR were cloned into the pTLN vector for expression in *X. laevis* oocytes [68]. After linearization of the plasmid DNA by MluI, capped complementary RNA was transcribed with the mMessage mMachine kit (Ambion) and purified with the RNeasy kit (Qiagen). For expression, 1–50 ng of RNA was injected into defolliculated oocytes. Two-electrode voltage clamp measurements were performed 1 d after injection at 20°C (OC-725B, Warner Instrument Corp.). Currents were recorded in bath solutions containing 10 mM HEPES (pH 7), 130 mM NaCl, and the indicated concentrations of cysteamine and divalent cations. In case of solutions containing Zn^2+^, cysteamine was replaced by propylamine. The membrane potential in all dose–response measurements was set to −40 mV. As ELIC is permeable to divalent cations, we tested if endogenous calcium-activated chloride channels affected our measurements. To chelate intracellular calcium ions, the oocytes were incubated for 15 to 30 min in bath solutions lacking divalent ions but containing 10 µM BAPTA-AM. Dose–response curves in the presence of calcium obtained from BAPTA-AM-treated oocytes did not differ from the measurements of the untreated oocytes even at elevated Ca^2+^ concentration (Figure S6). The lack of a significant effect is likely due to the strong outward-rectification of calcium-activated chloride channels, which do not pass significant currents at negative voltages.

### Patch Clamp Recording in *X. oocytes*

*X. laevis* oocytes were transferred to a hyperosmotic solution to manually remove the vitelline layer. Membrane patches were recorded in the excised outside-out configuration 3–5 d after injection of mRNA with an Axopatch 200B amplifier (Axon Instruments) at 20°C. Data were sampled at 100 µs, filtered with 1,000 Hz, and analyzed using Clampfit (Axon Instruments, Inc.). Bath solutions contained 10 mM HEPES (pH 7.0), 150 mM NaCl, and indicated concentrations of ligands and divalent cations. Electrodes had a resistance of 3–5 MΩ. Pipette solutions contained 150 mM NaCl, 10 mM EGTA, 5 mM MgCl_2_, and 10 mM HEPES at pH 7.0. Bath electrodes were placed in 1 M KCl solution connected to the bath solution by Agar bridges. The agonists were applied to the patch using a stepper motor (SF77B Perfusion fast step, Warner).

### Patch Clamp Recording in HEK 293 Cells

Human embryonic kidney 293 cells (American Type Culture Collection-CRL-1573;LGC Promochem) were maintained at 37°C in a 95% air/5% CO_2_ incubator in DMEM supplemented with 0.11 g/l sodium pyruvate, 10% (v/v) heat-inactivated fetal bovine serum, 100 U/ml penicillin G, 100 µg/ml streptomycin sulfate, and 2 mM L-glutamine (Invitrogen). Cells (passaged every 2 d, up to 30 times) were plated and transfected by calcium phosphate-DNA coprecipitation [69], with a total amount of DNA of 3 µg/dish (82% ELIC and 18% eGFP DNA, both subcloned in pcDNA3).

Cells were bathed in an extracellular solution containing (mM): 150 KCl, 0.05 or 0.2 CaCl_2_, and 10 HEPES, pH adjusted to 7.4 with KOH (osmolarity 310 mOsm). Patch pipettes were pulled from thick-walled borosilicate glass (GC150F; Harvard Apparatus) and fire polished to a resistance of 8–12 MΩ. Intracellular solution contained (mM): 150 KCl, 0.5 CaCl_2_, 5 EGTA, and 10 HEPES, pH adjusted to 7.4 with KOH. Agonist-evoked currents were recorded at 20°C with an Axopatch 200B amplifier (Molecular Devices) from outside-out patches held at −100 mV. Patches were stepped to this holding voltage 0.2 s before the agonist was applied and otherwise held at −40 mV. No correction for junction potential was applied (calculated value 0.2 mV). Currents were filtered at 5 kHz, digitized at 50 kHz with Digidata 1322A, and saved directly on computer with Clampex software (all MDS Analytical Technologies).

All concentration jumps were performed using a piezo stepper (Burleigh instruments) with an application tool made from theta tube glass (Hilgenberg; final tip diameter, 150 µm). Voltage commands for the piezo stepper were 200 ms square pulses conditioned by low-pass eight-pole Bessel filtering (−3 dB frequency 5 kHz) to smooth oscillations. Actual exchange time was estimated by recording the open-tip response to the application of diluted extracellular solution (70% water) after rupture of the patch. Only patches in which the 20%–80% exchange time was faster than 250 µs were included in the analysis.

Agonist solutions were freshly prepared every day from 1 M stock solutions. Propylamine was applied at a concentration known to elicit maximum response (20 mM and 50 mM, for 50 and 200 µM Ca^2+^, respectively). Traces shown are averages of 5 or 10 individual agonist currents, separated by at least 10 s. Responses were averaged, and the time course of activation and deactivation (between 95% and 5% of the peak current level) was fitted with one exponential component (program Clampfit 9.0).

### Accession Code

The coordinates of the P4_3_ crystal form of ELIC in complex with Ba^2+^ have been deposited with the Protein Data Bank under code 2yn6.

## Supporting Information

Figure S1 Dose–response relationships at high Ca^2+^ concentrations. Cysteamine dose–response relationships of ELIC in the presence of different concentrations of Ca^2+^. Currents were recorded at −40 mV. The data are averages from at least 5 oocytes; errors are SD. The solid lines show fits to a Hill equation. (B) Schild plot quantifying the inhibition by Ca^2+^. EC_50_ values were obtained from fits to data shown in panel (A). Potencies of the antagonists (pA values) were obtained by linear regression; the intersection with the *x*-axis is indicated (•).
(JPG)

Figure S2 Barium binding. (A) Plot of maximum agonist responses and single channel currents at different Ba^2+^ concentrations. The currents are normalized to the control values (in the absence of Ba^2+^). Maximum cysteamine currents (blue symbols) were measured with the two-electrode voltage clamp technique. Single channel currents (green symbols) were measured from excised patches in the outside-out configuration. (B, C) Structure of the divalent cation binding site S_out_. Stereo representations of the binding region in two different crystal forms. The protein is shown as Cα-trace with selected side-chains close to Ba^2+^ (red sphere) shown as sticks. 2F_o_-F_c_ electron densities are shown as cyan mesh. The refined models used to calculate phases did not contain Ba^2+^-ions. (B) Space group P4_3_. The 2F_o_-F_c_ electron density was calculated at 3.3 Å and contoured at 1 σ. The anomalous difference electron density (calculated at 5 Å and contoured at 5 σ) was obtained from the same dataset. (C) Space group P2_1_. The 2F_o_-F_c_ electron density was calculated at 3.8 Å and contoured at 1 σ. The anomalous difference electron density (calculated at 5 Å and contoured at 5 σ) was obtained from the same dataset.
(JPG)

Figure S3 Divalent ion inhibition in mutants of the regulatory site S_out_. Dose–response relationships of the ELIC mutants D113N (A), D158N (B), and E150Q (C) activated by cysteamine at different concentrations of Ca^2+^ are shown. (D) Schild plots quantifying the inhibition of ELIC mutants by Ca^2+^. (E) Dose–response relationships of the ELIC double mutant D113A/D158A activated by cysteamine at different concentrations of Ba^2+^ are shown. (F) Schild plot quantifying the inhibition of the ELIC double mutant D113A/D158A by Ba^2+^. EC_50_ values were obtained from data shown in panels (A–C) and (E). Potencies of the antagonists (pA values) in (D) and (F) were obtained by linear regression; the intersection with the *x*-axis is indicated (•). WT is shown for comparison. The data presented in panels (A–C) and (E) are averages from at least 5 oocytes; errors are SD. The solid lines show fits to a Hill equation. Currents were recorded at −40 mV. A dose–response curve of WT in the absence of Ca^2+^ (dashed line) is shown for comparison.
(JPG)

Figure S4 Divalent ion inhibition in mutants of histidine residues in the extracellular domain. Dose–response relationships of the ELIC mutants H168A (A) and H176A (B) activated by cysteamine at different concentrations of Ca^2+^ are shown. (C) Schild plots quantifying the inhibition of ELIC mutants by Ca^2+^. EC_50_ values were obtained from data shown in panels (A–B). Potencies of the antagonists (pA values) were obtained by linear regression; the intersection with the *x*-axis is indicated (•). WT is shown for comparison. The data presented in panels (A–B) are averages from at least 5 oocytes; errors are SD. The solid lines show fits to a Hill equation. Currents were recorded at −40 mV. A dose–response curve of WT in the absence of Ca^2+^ (dashed line) is shown for comparison.
(JPG)

Figure S5 Inhibition by tetramethylammonium (TMA). Dose–response relationships of WT (A) and the mutant R91A (B) activated by cysteamine at different concentrations of TMA are shown. A dose–response curve of WT in the absence of TMA (dashed line) is shown for comparison. (C) Schild plots quantifying the inhibition by TMA. EC_50_ values were obtained from data shown in panels (A–B). Potencies of the antagonists (pA values) were obtained by linear regression; the intersection with the *x*-axis is indicated (•). WT is shown for comparison. The data presented in panels (A–B) are averages from at least 5 oocytes; errors are SD. The solid lines show fits to a Hill equation. Currents were recorded at −40 mV.
(JPG)

Figure S6 Ca^2+^ inhibition measured from BAPTA-AM-treated oocytes. Dose–response relationships of WT activated by cysteamine at different concentrations of Ca^2+^ are shown. To chelate intracellular Ca^2+^, oocytes were incubated in solutions lacking divalent ions but containing 10 µM Bapta-AM. (C) Schild plots comparing the inhibition of ELIC in BAPTA-treated oocytes by Ca^2+^. EC_50_ values were obtained from data shown in panel (A). Potencies of the antagonists (pA values) were obtained by linear regression; the intersection with the *x*-axis is indicated (•). WT from oocytes treated by standard procedures is shown for comparison. The data presented in panel (A) are averages from at least 5 oocytes; errors are SD. The solid lines show fits to a Hill equation. Currents were recorded at −40 mV.
(JPG)

Table S1 Dose–response relationships of agonists in the presence of different modulators.
(DOC)

Text S1 Supplementary discussion.
(DOCX)

## Abbreviations

5HT_3_R: serotonin receptor
GABAR: gaba receptor
GlyR: glycine receptor
nAChR: nicotinic acetylcholine receptor
pLGIC: pentameric ligand-gated ion channel
