# Supplementary figures and images for: Inhibition of the Prokaryotic Pentameric Ligand-Gated Ion Channel ELIC by Divalent Cations
Source: PLoS Biol. 2012 Nov 20;10(11):e1001429. doi: 10.1371/journal.pbio.1001429 (PMC3502511; doi:10.1371/journal.pbio.1001429)

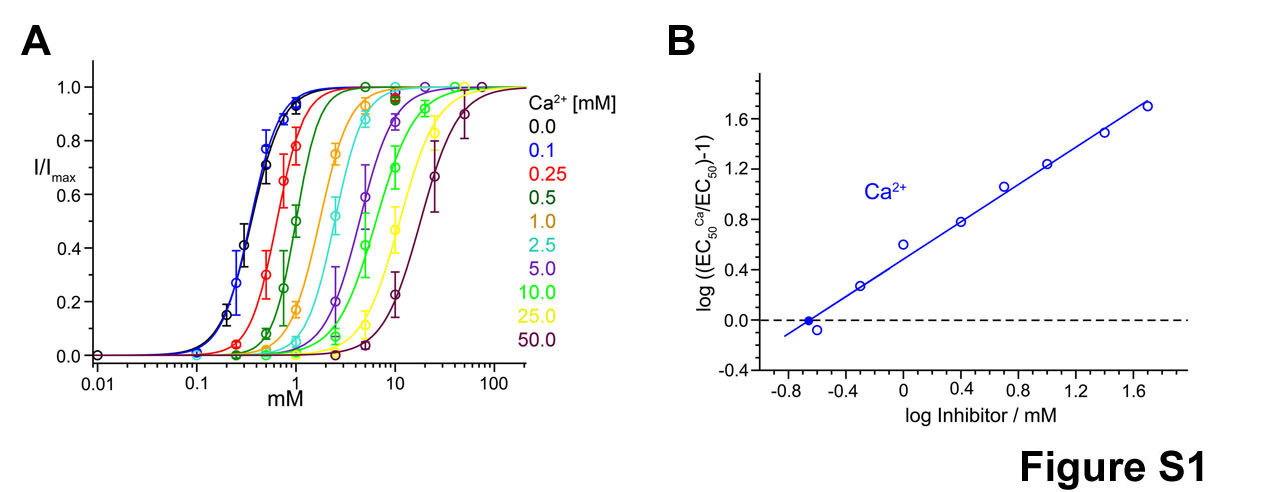

Supplement: Figure S1 — Dose–response relationships at high Ca2+ concentrations. Cysteamine dose–response relationships of ELIC in the presence of different concentrations of Ca2+. Currents were recorded at −40 mV. The data are averages from at least 5 oocytes; errors are SD. The solid lines show fits to a Hill equation. (B) Schild plot quantifying the inhibition by Ca2+. EC50 values were obtained from fits to data shown in panel (A). Potencies of the antagonists (pA values) were obtained by linear regression; the intersection with the x-axis is indicated (•). (JPG) [file pbio.1001429.s001.jpg]

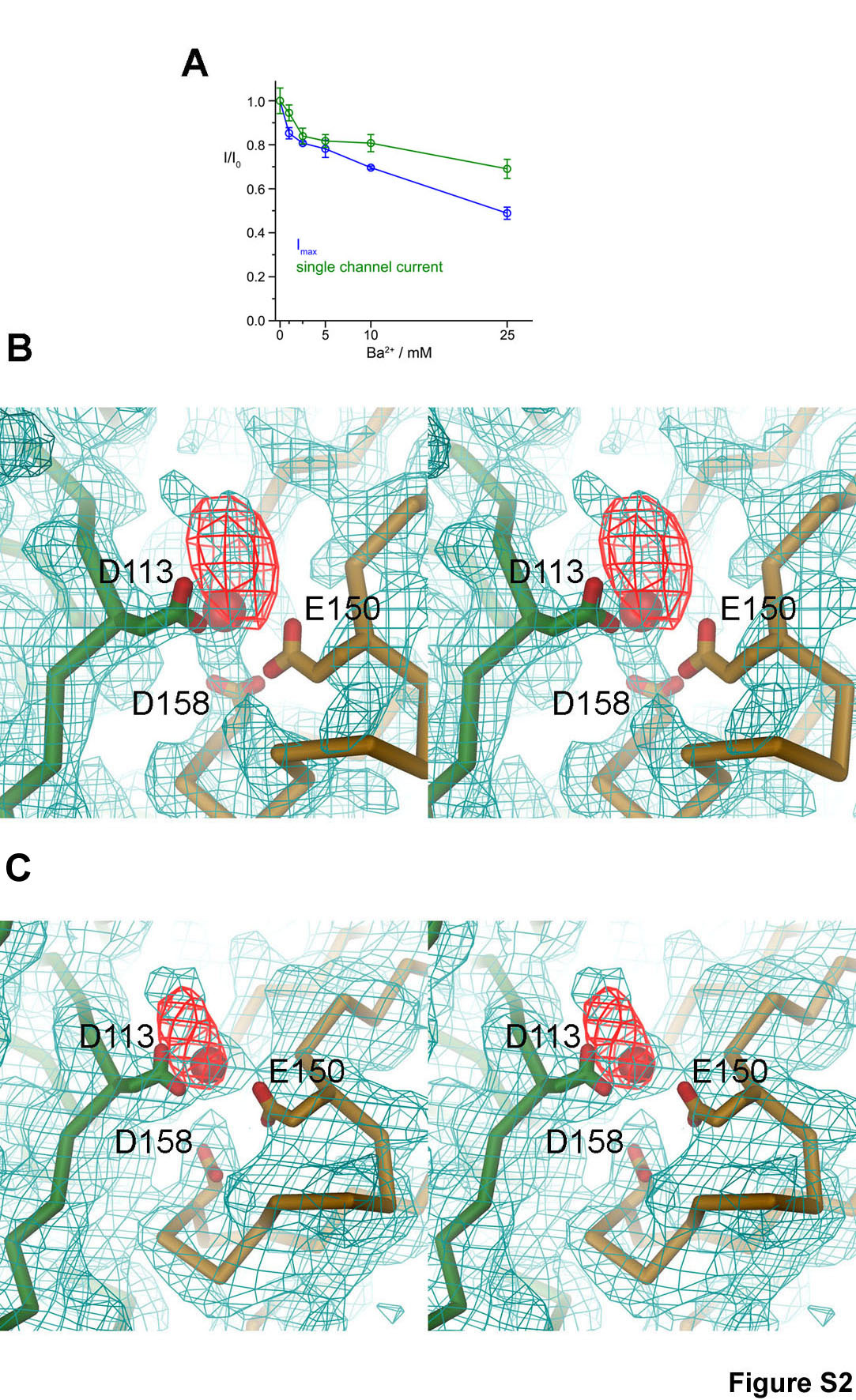

Supplement: Figure S2 — Barium binding. (A) Plot of maximum agonist responses and single channel currents at different Ba2+ concentrations. The currents are normalized to the control values (in the absence of Ba2+). Maximum cysteamine currents (blue symbols) were measured with the two-electrode voltage clamp technique. Single channel currents (green symbols) were measured from excised patches in the outside-out configuration. (B, C) Structure of the divalent cation binding site Sout. Stereo representations of the binding region in two different crystal forms. The protein is shown as Cα-trace with selected side-chains close to Ba2+ (red sphere) shown as sticks. 2Fo-Fc electron densities are shown as cyan mesh. The refined models used to calculate phases did not contain Ba2+-ions. (B) Space group P43. The 2Fo-Fc electron density was calculated at 3.3 Å and contoured at 1 σ. The anomalous difference electron density (calculated at 5 Å and contoured at 5 σ) was obtained from the same dataset. (C) Space group P21. The 2Fo-Fc electron density was calculated at 3.8 Å and contoured at 1 σ. The anomalous difference electron density (calculated at 5 Å and contoured at 5 σ) was obtained from the same dataset. (JPG) [file pbio.1001429.s002.jpg]

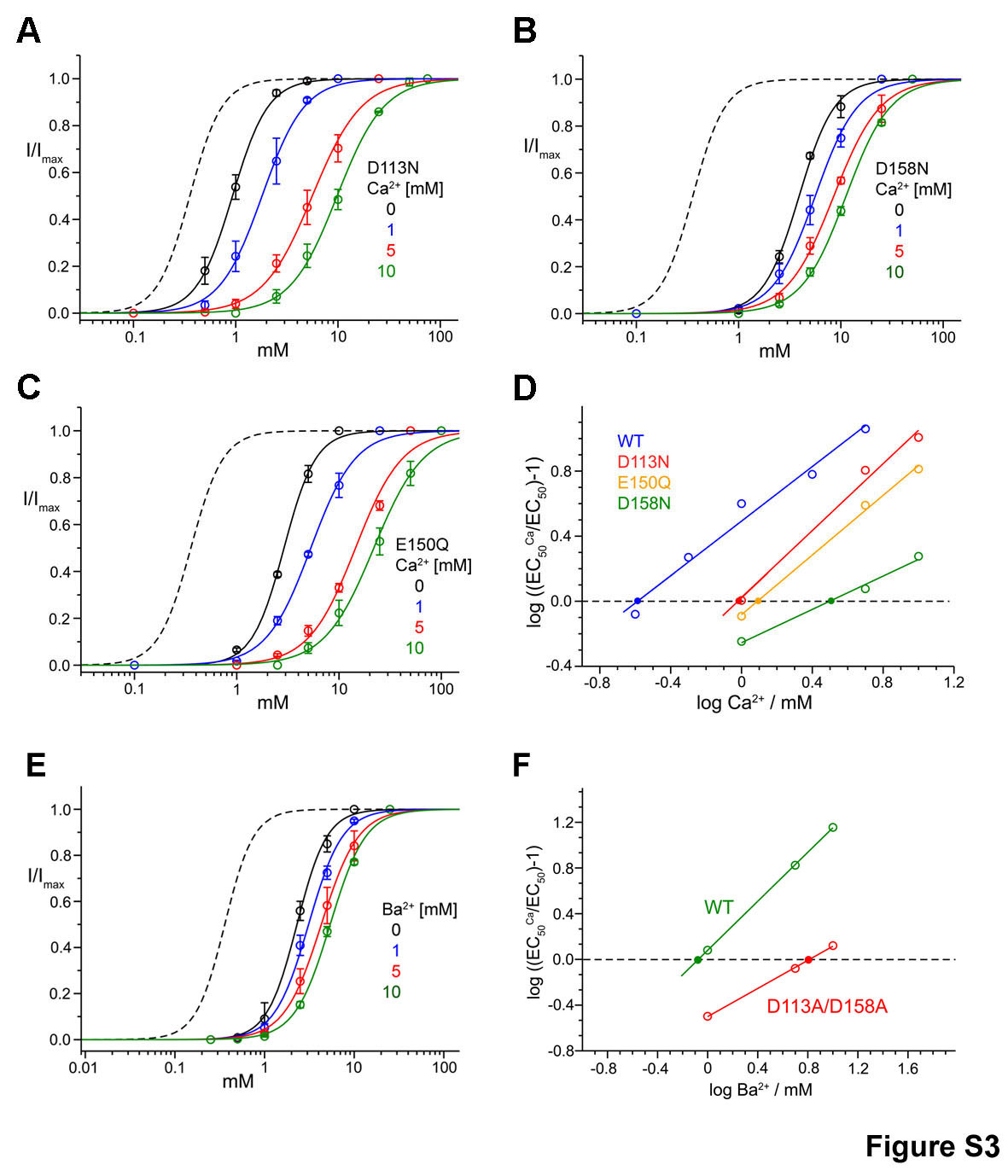

Supplement: Figure S3 — Divalent ion inhibition in mutants of the regulatory site Sout. Dose–response relationships of the ELIC mutants D113N (A), D158N (B), and E150Q (C) activated by cysteamine at different concentrations of Ca2+ are shown. (D) Schild plots quantifying the inhibition of ELIC mutants by Ca2+. (E) Dose–response relationships of the ELIC double mutant D113A/D158A activated by cysteamine at different concentrations of Ba2+ are shown. (F) Schild plot quantifying the inhibition of the ELIC double mutant D113A/D158A by Ba2+. EC50 values were obtained from data shown in panels (A–C) and (E). Potencies of the antagonists (pA values) in (D) and (F) were obtained by linear regression; the intersection with the x-axis is indicated (•). WT is shown for comparison. The data presented in panels (A–C) and (E) are averages from at least 5 oocytes; errors are SD. The solid lines show fits to a Hill equation. Currents were recorded at −40 mV. A dose–response curve of WT in the absence of Ca2+ (dashed line) is shown for comparison. (JPG) [file pbio.1001429.s003.jpg]

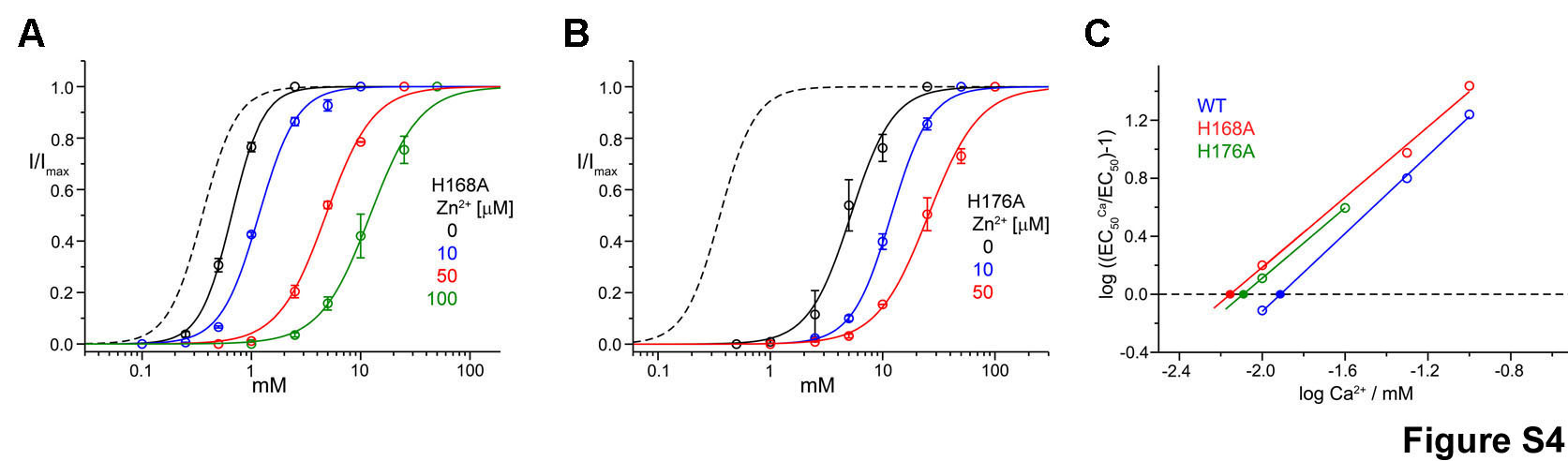

Supplement: Figure S4 — Divalent ion inhibition in mutants of histidine residues in the extracellular domain. Dose–response relationships of the ELIC mutants H168A (A) and H176A (B) activated by cysteamine at different concentrations of Ca2+ are shown. (C) Schild plots quantifying the inhibition of ELIC mutants by Ca2+. EC50 values were obtained from data shown in panels (A–B). Potencies of the antagonists (pA values) were obtained by linear regression; the intersection with the x-axis is indicated (•). WT is shown for comparison. The data presented in panels (A–B) are averages from at least 5 oocytes; errors are SD. The solid lines show fits to a Hill equation. Currents were recorded at −40 mV. A dose–response curve of WT in the absence of Ca2+ (dashed line) is shown for comparison. (JPG) [file pbio.1001429.s004.jpg]

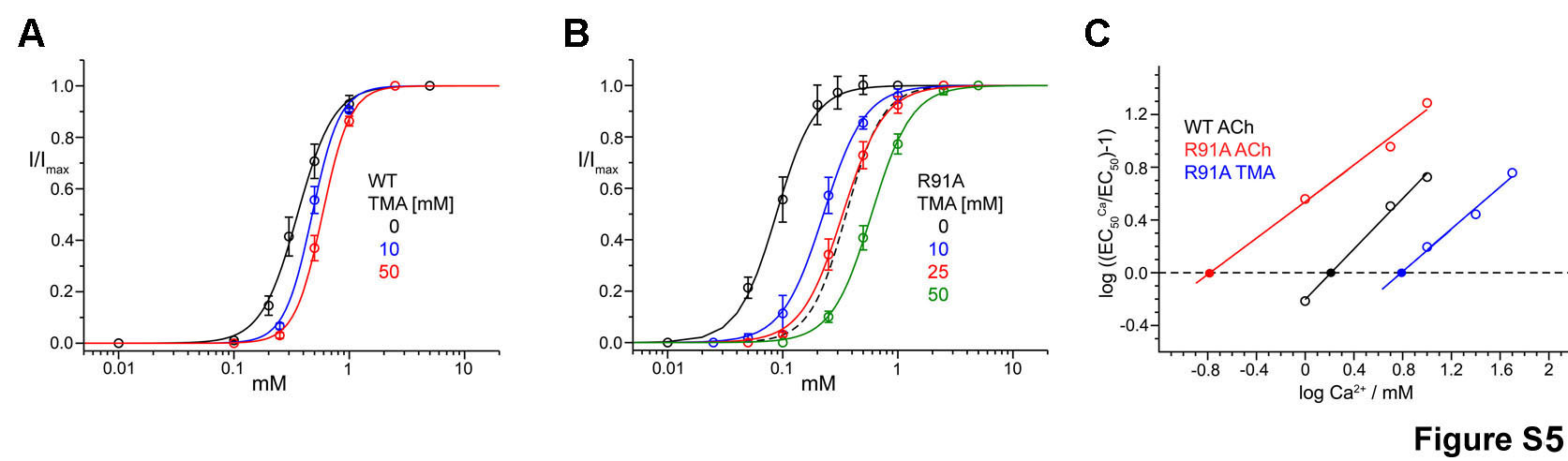

Supplement: Figure S5 — Inhibition by tetramethylammonium (TMA). Dose–response relationships of WT (A) and the mutant R91A (B) activated by cysteamine at different concentrations of TMA are shown. A dose–response curve of WT in the absence of TMA (dashed line) is shown for comparison. (C) Schild plots quantifying the inhibition by TMA. EC50 values were obtained from data shown in panels (A–B). Potencies of the antagonists (pA values) were obtained by linear regression; the intersection with the x-axis is indicated (•). WT is shown for comparison. The data presented in panels (A–B) are averages from at least 5 oocytes; errors are SD. The solid lines show fits to a Hill equation. Currents were recorded at −40 mV. (JPG) [file pbio.1001429.s005.jpg]

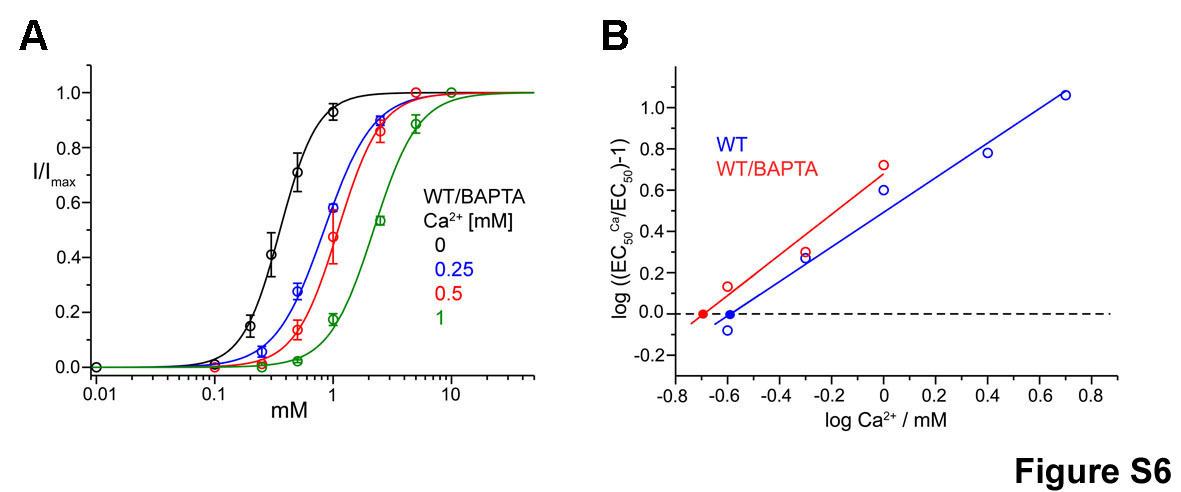

Supplement: Figure S6 — Ca2+ inhibition measured from BAPTA-AM-treated oocytes. Dose–response relationships of WT activated by cysteamine at different concentrations of Ca2+ are shown. To chelate intracellular Ca2+, oocytes were incubated in solutions lacking divalent ions but containing 10 µM Bapta-AM. (C) Schild plots comparing the inhibition of ELIC in BAPTA-treated oocytes by Ca2+. EC50 values were obtained from data shown in panel (A). Potencies of the antagonists (pA values) were obtained by linear regression; the intersection with the x-axis is indicated (•). WT from oocytes treated by standard procedures is shown for comparison. The data presented in panel (A) are averages from at least 5 oocytes; errors are SD. The solid lines show fits to a Hill equation. Currents were recorded at −40 mV. (JPG) [file pbio.1001429.s006.jpg]
