# Supplementary material for: Inhibition of the Prokaryotic Pentameric Ligand-Gated Ion Channel ELIC by Divalent Cations
Source: PLoS Biol. 2012 Nov 20;10(11):e1001429. doi: 10.1371/journal.pbio.1001429 (PMC3502511; doi:10.1371/journal.pbio.1001429)
Supplement: Table S1 — Dose–response relationships of agonists in the presence of different modulators. (DOC) [file pbio.1001429.s007.doc]

**Table S1** Dose-response relationships of agonists in the presence of different modulators

|  |  | [mM] | EC_50_^*^ | n |  |  | [mM] | EC_50_^*^ | n |
| --- | --- | --- | --- | --- | --- | --- | --- | --- | --- |
| WT | Ca^2+^ | 0 | 0.36 | 2.8 | WT | Ca^2+^ | 0.1 | 0.35 | 2.9 |
| WT | Ca^2+^ | 0.25 | 0.64 | 3.2 | WT | Ca^2+^ | 0.5 | 1.00 | 2.9 |
| WT | Ca^2+^ | 1.0 | 1.73 | 2.9 | WT | Ca^2+^ | 2.5 | 2.46 | 3.0 |
| WT | Ca^2+^ | 5.0 | 4.37 | 2.5 |  |  |  |  |  |
| WT/BAPTA | Ca^2+^ | 0 | 0.36 | 2.7 | WT/BAPTA | Ca^2+^ | 0.25 | 0.84 | 2.1 |
| WT/BAPTA | Ca^2+^ | 0.5 | 1.07 | 2.4 | WT/BAPTA | Ca^2+^ | 1.0 | 2.23 | 2.3 |
| WT | ACh | 1 | 0.57 | 2.5 | WT | ACh | 5.0 | 1.49 | 2.4 |
| WT | ACh | 10.0 | 2.25 | 2.5 |  |  |  |  |  |
| WT/0.25Ca^2+^ | ACh | 0 | 0.64 | 3.2 | WT/0.25Ca^2+^ | ACh | 1 | 1.16 | 1.9 |
| WT/0.25Ca^2+^ | ACh | 5 | 3.46 | 2.4 | WT/0.25Ca^2+^ | ACh | 10 | 5.08 | 2.1 |
| WT/0.5Ca^2+^ | ACh | 0 | 1.00 | 3.7 | WT/0.5Ca^2+^ | ACh | 1 | 1.83 | 2.5 |
| WT/0.5Ca^2+^ | ACh | 5 | 4.03 | 2.2 | WT/0.5Ca^2+^ | ACh | 10 | 8.45 | 2.7 |
| WT/1Ca^2+^ | ACh | 0 | 1.73 | 2.8 | WT/1Ca^2+^ | ACh | 1 | 2.68 | 2.0 |
| WT/1Ca^2+^ | ACh | 5 | 5.12 | 2.4 | WT/1Ca^2+^ | ACh | 10 | 8.24 | 1.9 |
| WT | Zn^2+^ | 0.01 | 0.92 | 3.2 | WT | Zn^2+^ | 0.05 | 3.72 | 2.2 |
| WT | Zn^2+^ | 0.1 | 10.1 | 2.3 |  |  |  |  |  |
| WT | Ba^2+^ | 1 | 0.76 | 3.7 | WT | Ba^2+^ | 5 | 2.68 | 2.3 |
| WT | Ba^2+^ | 10 | 5.27 | 2.3 |  |  |  |  |  |
| WT | Sr^2+^ | 1 | 0.75 | 2.9 | WT | Sr^2+^ | 5 | 2.11 | 2.7 |
| WT | Sr^2+^ | 10 | 3.41 | 2.3 |  |  |  |  |  |
| WT | Mg^2+^ | 1 | 1.04 | 3.1 | WT | Mg^2+^ | 5 | 3.05 | 2.4 |
| WT | Mg^2+^ | 10 | 5.31 | 2.1 |  |  |  |  |  |
| D86A | Ca^2+^ | 0 | 0.57 | 2.3 | D86A | Ca^2+^ | 1 | 2.30 | 2.1 |
| D86A | Ca^2+^ | 5 | 5.31 | 1.9 | D86A | Ca^2+^ | 10 | 11.2 | 2.9 |
| S84A | Ca^2+^ | 0 | 0.36 | 2.5 | S84A | Ca^2+^ | 1 | 1.55 | 1.6 |
| S84A | Ca^2+^ | 5 | 2.74 | 1.9 | S84A | Ca^2+^ | 10 | 5.43 | 1.4 |
| N251A | Ca^2+^ | 0 | 0.29 | 2.3 | N251A | Ca^2+^ | 1 | 0.84 | 3.5 |
| N251A | Ca^2+^ | 5 | 1.90 | 2.7 | S84A | Ca^2+^ | 10 | 5.31 | 1.8 |
| E150A | Ca^2+^ | 0 | 1.40 | 2.4 | E150A | Ca^2+^ | 1 | 2.20 | 2.6 |
| E150A | Ca^2+^ | 5 | 6.10 | 2.0 | E150A | Ca^2+^ | 10 | 8.99 | 1.9 |
| E150Q | Ca^2+^ | 0 | 2.92 | 2.8 | E150Q | Ca^2+^ | 1 | 5.29 | 2.0 |
| E150Q | Ca^2+^ | 5 | 14.6 | 1.9 | E150Q | Ca^2+^ | 10 | 21.9 | 1.8 |
| D113A | Ca^2+^ | 0 | 0.95 | 2.6 | D113A | Ca^2+^ | 1 | 0.88 | 2.2 |
| D113A | Ca^2+^ | 2.5 | 1.46 | 1.8 | D113A | Ca^2+^ | 5 | 1.86 | 1.9 |
| D113A | Ca^2+^ | 10 | 2.90 | 1.9 | D113A | Ca^2+^ | 25 | 6.37 | 1.5 |
| D113N | Ca^2+^ | 0 | 0.92 | 2.5 | D113N | Ca^2+^ | 1 | 1.81 | 2.1 |
| D113N | Ca^2+^ | 5 | 5.59 | 1.8 | D113N | Ca^2+^ | 10 | 9.78 | 1.9 |
| D158A | Ca^2+^ | 0 | 1.78 | 2.6 | D158A | Ca^2+^ | 1 | 1.79 | 3.2 |
| D158A | Ca^2+^ | 5 | 3.33 | 3.1 | D158A | Ca^2+^ | 10 | 5.43 | 2.0 |
| D158A | Ca^2+^ | 20 | 9.38 | 2.0 |  |  |  |  |  |
| D158N | Ca^2+^ | 0 | 3.88 | 2.5 | D158N | Ca^2+^ | 1 | 5.61 | 2.1 |
| D158N | Ca^2+^ | 5 | 8.50 | 1.9 | D158N | Ca^2+^ | 10 | 11.2 | 2.0 |
| 113A/158A | Ca^2+^ | 0 | 2.33 | 2.6 | 113A/158A | Ca^2+^ | 1 | 1.59 | 3.5 |
| 113A/158A | Ca^2+^ | 5 | 1.81 | 2.9 | 113A/158A | Ca^2+^ | 10 | 2.96 | 2.6 |
| 113A/158A | Ca^2+^ | 25 | 3.35 | 2.4 |  |  |  |  |  |
| 113A/158A | Zn^2+^ | 0 | 3.70 | 1.4 | 113A/158A | Zn^2+^ | 0.01 | 4.56 | 2.0 |
| 113A/158A | Zn^2+^ | 0.1 | 4.22 | 1.7 |  |  |  |  |  |
|  |  | [mM] | Ec50 | n |  |  | [mM] | Ec50 | n |
| 113A/158A | Ba^2+^ | 1 | 3.07 | 2.3 | 113A/158A | Ba^2+^ | 5 | 3.07 | 2.1 |
| 113A/158A | Ba^2+^ | 10 | 5.42 | 2.2 |  |  |  |  |  |
| R91A | Ca^2+^ | 0 | 0.09 | 2.6 | R91A | Ca^2+^ | 0.5 | 0.24 | 1.7 |
| R91A | Ca^2+^ | 1 | 0.41 | 1.8 | R91A | Ca^2+^ | 5 | 0.92 | 1.7 |
| R91A | ACh | 1 | 0.40 | 3.2 | R91A | ACh | 5 | 0.88 | 3.3 |
| R91A | ACh | 10 | 1.78 | 2.7 |  |  |  |  |  |
| R91A | TMA | 10 | 0.23 | 2.4 | R91A | TMA | 25 | 0.33 | 2.4 |
| R91A | TMA | 50 | 0.59 | 2.5 |  |  |  |  |  |
| H168A | Zn^2+^ | 0 | 0.67 | 3.0 | H168A | Zn^2+^ | 0.01 | 1.17 | 2.5 |
| H168A | Zn^2+^ | 0.05 | 4.82 | 2.1 | H168A | Zn^2+^ | 0.1 | 12.2 | 1.9 |
| H176A | Zn^2+^ | 0 | 5.16 | 2.3 | H176A | Zn^2+^ | 0.01 | 11.9 | 2.5 |
| H176A | Zn^2+^ | 0.025 | 25.5 | 1.9 |  |  |  |  |  |

Propylamine was used as agonist in experiments involving Zn^2+^, cysteamine in all other cases.

*EC_50_ in mM
