# Supplementary material for: Inhibition of the Prokaryotic Pentameric Ligand-Gated Ion Channel ELIC by Divalent Cations
Source: PLoS Biol. 2012 Nov 20;10(11):e1001429. doi: 10.1371/journal.pbio.1001429 (PMC3502511; doi:10.1371/journal.pbio.1001429)
Supplement: Text S1 — Supplementary discussion. (DOCX) [file pbio.1001429.s008.docx]

***Supplementary Discussion - The mechanism of the action of divalent ions on ELIC***

*The experimental data we have to explain*

By binding to S_out_, extracellular calcium produces several, apparently disparate effects on functional ELIC responses. These effects are additional to the reduction in single channel conductance and independent of it.

These modulatory effects of calcium include:

1) a reduction in the potency of agonists, which results in a parallel shift to the right of the agonist dose-response curve. This effect appears at low calcium concentration (0.1-1 mM) and is not associated with changes in the agonist maximum response.

2) at higher calcium concentrations (2-5 mM), there is further reduction in agonist potency, and this is associated with a pronounced decrease in the agonist maximum response (about 50% at 6 mM –channel block subtracted).

3) low calcium does not affect how fast ELIC channel open when they are saturated by agonist, but increases the concentration of agonist needed to reach this “limiting” rate of opening.

4) the decreases in agonist potency produced by calcium and those produced by the competitive antagonist acetylcholine are independent, namely they are unaffected by the presence or absence of the other.

*The effects of calcium on the agonist dose-response relation can be explained by a decrease in efficacy within a simple del Castillo-Katz activation mechanism*

The simplest scheme for the activation of a ligand-gated ion channel was proposed by Jose’ del Castillo and Bernard Katz in 1957[1]. In this scheme


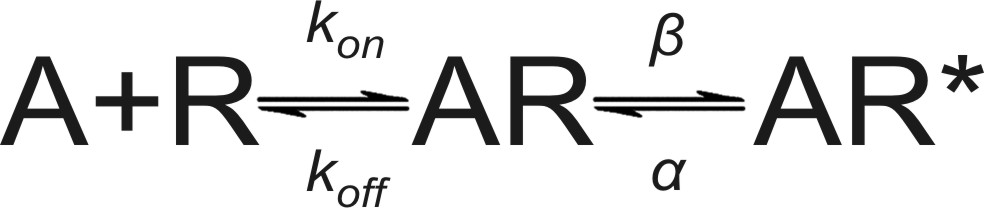


(1)

activation involves two steps. The first is the binding of the agonist A to the channel R, with microscopic affinity described by the dissociation constant *K*_d_ (itself the ratio between the dissociation rate constant *k*off and the association rate constant *k*_on_). The second step is a conformational change, where the agonist-bound resting receptor (AR) opens to the conducting form AR*. The position of the equilibrium between AR and AR* is described by an equilibrium constant *E,* that reflects agonist efficacy and is the ratio between the opening rate constant *β* and the closing rate constant *α*.

More complex schemes are needed to explain all the aspects of channel functional behaviour, and such schemes incorporate the existence of more than one agonist binding site, the possibility of unliganded and partially-liganded openings, intermediate states in the conformational change and desensitisation. Nevertheless, the del Castillo-Katz scheme is sufficient to describe much of basic channel pharmacology (reviewed in ref. [2] and exploring it gives useful insight.

Indeed, if we look only at the ELIC equilibrium responses (ie the agonist dose-response curves), all the effects of calcium can be explained within the del Castillo-Katz mechanism, just by assuming 1) that calcium decreases agonist efficacy *E* and2) that full agonist efficacy *E* is high in ELIC.

The value of efficacy *E*

The second assumption appears to be quite plausible. In the del Castillo-Katz mechanism, the channel open probability is given by

(2)


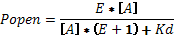

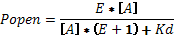


Maximum open probability (*P*_open_) is

(3)


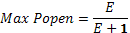

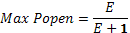


and the *EC*_50_ for the agonist is

(4)


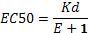

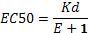


Figure 1A shows that cysteamine opens ELIC channels with a high maximum open probability. Our single channel data (Marabelli et al., in preparation) suggest that the maximum *P*_open_ reaches 98%. From Equation 3, this implies that agonist efficacy is of the order of 50 (50/51= 98%). This value is not dissimilar to that seen for other members of the superfamily, such as the nicotinic ACh receptor and the glycine receptor, when they are activated by full agonists.

The effects of decreasing efficacy

When efficacy *E* is this high, the effects of a small decrease in *E* on the maximum agonist response are hard to detect in practice. For instance, a two-fold decrease in *E* would reduce maximum open probability from 98% to 96% (that is from 50/51 to 25/26).

Even a two-fold decrease in *E* would however cause a detectable shift in agonist potency, as the agonist *EC*_50_ would almost double (see equation 4). This is in line with the observations at low calcium concentrations.

It is only when the decrease in *E* is much larger, and *E* drops to values below 10, that a decline in the maximum *P*_open_ becomes noticeable, as the maximum *P*_open_ falls below 90%. This is in line with our observations at higher calcium concentrations, where more of the calcium binding sites are occupied and calcium can have a greater effect on *E*.

Thus there is no need to hypothesize that low calcium and high calcium have different microscopic effects: a progressive decrease in agonist efficacy *E*, more marked at the higher calcium concentrations, is all that is needed to explain our observations.

*A more detailed activation mechanism is required to explain the lack of an effect of calcium on the limiting rate of channel gating*

In another set of experiments we produced ELIC currents by near-instantaneous applications of agonists (“concentration jumps”) to channels in outside-out patches. The time course with which these currents reach their steady-state value depends in principle on all the rate constants in the activation mechanism. However, if agonist is applied at concentrations that are very high and saturate the receptor, it can be shown that these maximum agonist current responses effectively increase as single exponentials with time constant

(5)


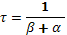

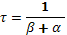


where *β* is the opening rate constant and *α* the closing rate constant [3,4].

We already know that efficacy *E* is high for ELIC. As efficacy is the ratio of *β* /*α*, the value of *β* must be much bigger than that of *α* (50-fold), and therefore the time constant is influenced mostly by the value of *β* .

If calcium decreases *E* (as we have to assume, in order to explain its effects on the agonist dose-response curves), it can do so either by decreasing *β* or by increasing *α*. Neither of these effects can be reconciled with our observations, if the del Castillo-Katz scheme is valid for our channel, because we did not observe a change in the limiting rate of gating or in the time course of the current at the end of the agonist pulse (deactivation). From the arguments above, it is clear that if there is a decrease in *β*, we should see a slowing of the maximum rate of current development and that this should be detectable even for fairly small changes in *β* . If, on the other hand calcium decreases *E* by increasing *α*, the limiting rate of gating would not change (given that *β* >>* α*), but speeding the channel closing rate *α* should result in a faster decline in the current when the agonist pulse ends, that is a speeding up of deactivation, which was not observed either.

Thus it may be that the del Castillo-Katz model is inadequate to explain the action of calcium. Indeed, it could be that activation of the channel requires not a single conformational change, but two. If the rate-limiting step for gating in the agonist jump experiments is not opening, but an earlier, slower step, calcium could still change efficacy and shift agonist potency and maximum responses *without* affecting the time course of the current in our agonist concentration jumps.

This is because, if reaching this reaction intermediate is slow enough, the agonist limiting relaxation will be dominated by this step, rather than by the opening itself. This explanation is qualitatively plausible because mechanisms incorporating reaction intermediates are necessary to explain the single channel kinetic properties of channels related to ELIC, such as nicotinic ACh receptors and glycine receptors, as shown by our own work and that of the groups of Sine and Auerbach (see main discussion, [5-7].

The simulation below is a proof of principle to show that this explanation is not only qualitatively plausible but also that it can work quantitatively, with at least one set of rate constant values. The solution is certainly not unique, and eq.6 is not proposed as a complete mechanism for the ELIC receptor. Nevertheless the rate constant values used are not unrealistic, as they are not far from our estimates from mechanism fitting of ELIC single channel data (Marabelli et al., in preparation) or from rate constants estimated from other pLGICs.

The simplest mechanism incorporating a reaction intermediate is

(6)


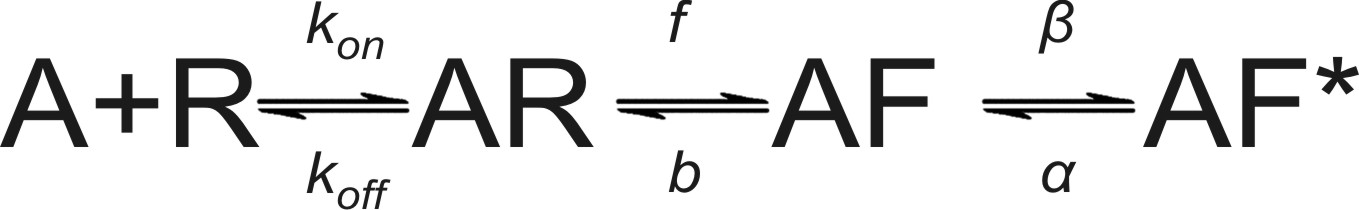


In this minimal “flip” mechanism, there is an additional state AF (for flipped), which is still closed but has changed conformation. The maximum *P*_open_ becomes

(7)


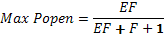

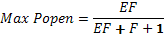


For the calculation of the time course of the saturating agonist responses, the mechanism can be simplified by pooling the states separated by a binding step (R and AR) into a single state [R,AR]. This can be done because we have a very high concentration of agonist A, making the association rate, *k*_on_ [A] very fast. For the same reason, the pooled state [R,AR] is almost entirely made of AR.

The mechanism then becomes

(8)


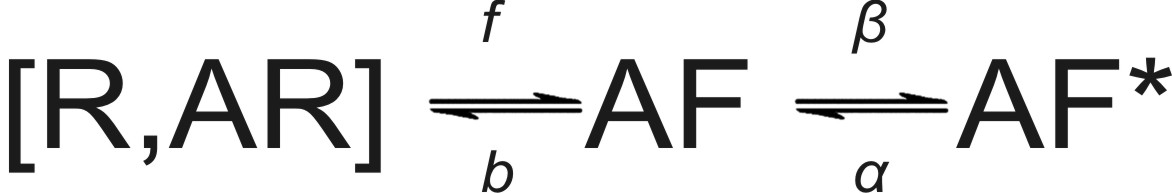


and has four rate constants: *β* (open),* α* (close), *f* (flip) and *b* (unflip) and the ratios of these give the equilibrium constants *E* = *β* /*α* and *F* = *f/b*. The values of the rate constants are constrained because the maximum *P*_open_ of the channel is high, suggesting that *β* >>* α* and *f* >=*b*.

We know also that the risetime of the current produced by the high concentration agonist jump has a time constant of 17-20 ms (this is considerably slower in ELIC than in other pLGICs).

The black trace in Figure 1A shows the current on-relaxation calculated for this mechanism. This is identical to a single exponential with a time constant of 20 ms (shown for reference as a red dashed curve). The values used in the calculation were *β* = 50000 s^-1^, *α* = 1000 s^-1^, *f* = *b*= 50 s^-1^. They make *E*= 50 and *F* = 1 and imply a maximum *P*_open_ of 96%.

Let’s now assume that we are in the presence of calcium and that calcium has reduced efficacy (by decreasing *β* ) by 5-fold. This would result, as discussed above, in a shift in the agonist potency and in a reduction in the maximum *P*_open_ (from 96% to 83%; in the model discussed in the next section, where calcium binding is explicit, this would be reached at approximately 1 mM calcium). However, as shown in Figure 1B, even this fairly strong effect of calcium has only a tiny effect on the risetime of the limiting agonist current (the calculated agonist current is shown in black and compared to a reference exponential curve with a time constant of 20 ms, red dashed curve). Note that for the purpose of showing some effect of calcium in the calculation, we have chosen to show the consequences of a reduction in efficacy greater than that likely to prevail in our concentration jump experiments (where we had no effect of calcium on the maximum response).

*More detailed mechanisms and the equilibrium effects of calcium*

The arguments above show that incorporating an intermediate state that limits the fastest rate of ELIC gating allows us to account for our observations in the agonist jump experiments. This more detailed mechanism can still explain the effects of calcium on the agonist dose-response curves, and in this section we will show that with an appropriate example.

If we incorporate in the flip mechanism the binding of calcium, we obtain

(9)


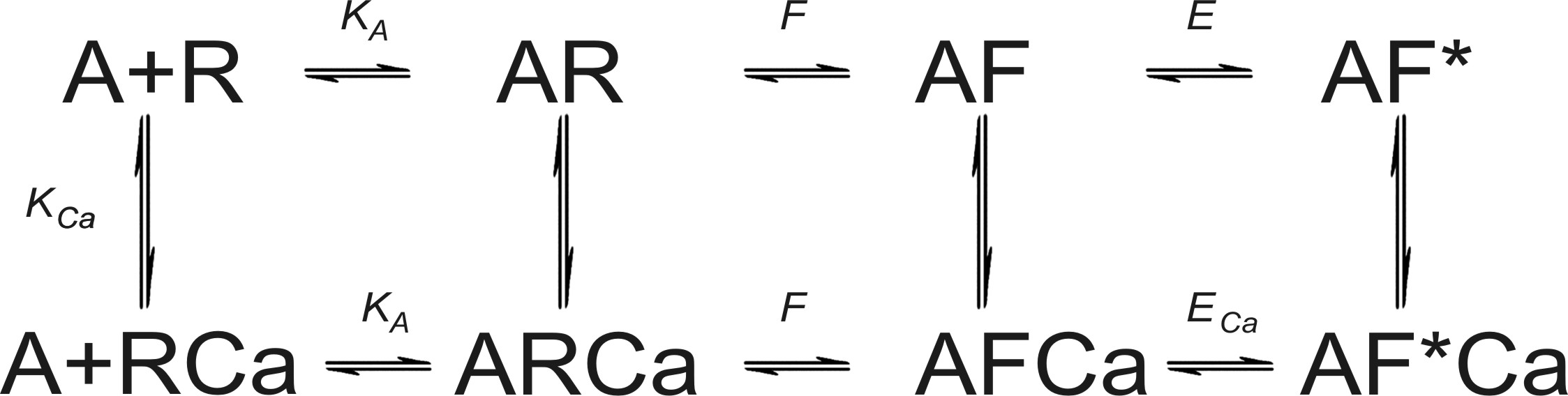


For clarity, the scheme above shows the names of the equilibrium constants for each step (rather than the rate constants as in schemes 1, 6 and 8). Note that *K*_A_ and *K*_Ca_ are the microscopic dissociation constants for the agonist A and for calcium, respectively. Calcium is assumed to affect only efficacy, which is reduced from *E* to *E*_Ca_ in the calcium-bound channel (bottom line of the mechanism).


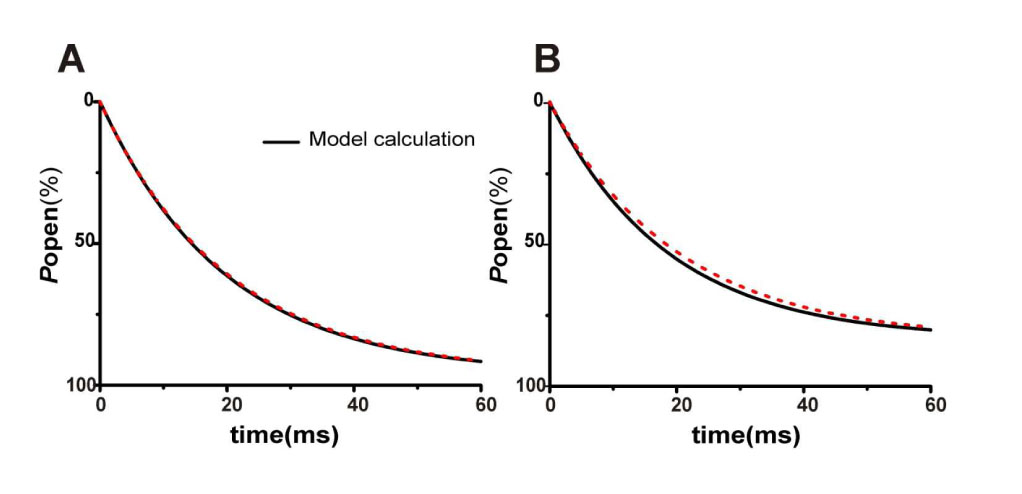


**Figure 1 Calcium can reduce efficacy and the maximum open probability of ELIC without changing the maximum rate of current onset, if this is limited by the channel’s slow access to an activation intermediate.** The black curves in the graphs are the calculated time course of currents produced by instantaneous applications of saturating agonist concentrations (at time 0, current expressed as open probability). The dashed red curves are single exponentials with time constant of 20 ms and the same maximum values as the currents and are shown for reference. Currents are calculated from mechanism (8). The values used in the control calculation (0 calcium, graph A) were *β* = 50000 s^-1^, *α* = 1000 s^-1^, *f* = *b*= 50 s^-1^ . Hence *E*= 50 and *F* = 1 (where efficacy *E* is the equilibrium constant for the final opening step and *F* is the equilibrium constant for access to the preceding intermediate state) and the maximum *P*_open_ is 96%. (B) Calcium can reduce the value of *β* by as much as 5-fold with negligible effects on the time course of current onset. Note that the steady-state value of this maximum current has decreased to a *P*_open_ value of 83%. The calculations with the more complete models in Figure 2 indicate that this reduction in maximum *P*_open_ is expected to occur at approximately 1 mM calcium, e.g. a concentration much greater than the one tested in the agonist jump experiments (0.2 mM).

Figure 2A shows the effect of progressively greater calcium concentrations on the dose-response curves for a full agonist. These effects are calculated from the scheme above, assuming that calcium reduces efficacy by up to 50-fold (when the channel is fully bound to calcium) and that *E* = 50 and *F* = 1. The latter values are chosen because the maximum *P*_open_ is high in control conditions.

It is clear from the Figure that the lowest calcium concentration (in this example half the calcium dissociation constant) shifts the agonist dose-response curve to the right, without changing the maximum much. Greater calcium concentrations (at least 5-fold the calcium dissociation constant) are required before the maximum response starts to decline. We can also plot the agonist dose-response curves after normalising them to their maximum response. This shows clearly the parallel shift to the right produced by calcium (Figure 2B).


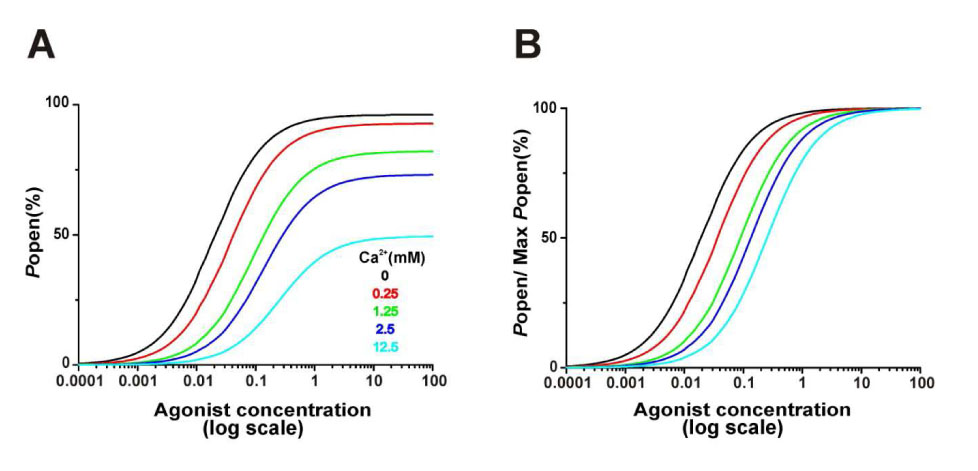


**Figure 2 The decrease in efficacy induced by calcium results in a reduction in agonist potency at low calcium and in an additional decrease in maximum response at high calcium.** The graphs show agonist concentration-response curves calculated from mechanism (9). This is a mechanism that incorporates an intermediate state in the activation of the channel. The calculations are based on values of *E*=50 and *F*=1. Calcium is assumed to bind to the resting state of the channel with a microscopic affinity of 0.25 mM (in line with the estimate from the Schild plot) and to reduce the value of efficacy by 50-fold when bound to the channel. The agonist concentration is expressed as a multiple of the (unknown) agonist microscopic dissociation constant (*K*_A_ in mechanism 9). In Figure 2A responses are expressed as absolute *P*_open_ values, and in Figure 2B as values normalised to the maximum agonist response.

*Plotting the effect of calcium as a Schild plot*

Schild analysis is used in pharmacology to characterise competitive antagonists, compounds whose binding is mutually exclusive with that of agonists. The dose ratios (e.g. the shifts) produced by the antagonist in the agonist dose-response curves are plotted in a Schild plot, as log(dose ratio-1) against the concentration of antagonist (on a log scale). If the antagonist is competitive, this produces a line with a slope of 1. The intercept between the line and the x-axis estimates the antagonist microscopic dissociation constant, a result that is at the basis of receptor characterization in classical pharmacology [8]. This result holds also for mechanisms that are more complicated that that for which the Schild analysis was originally derived [9]

It may appear surprising that we should see a Schild-like pattern for the effects of calcium, given our hypothesis that calcium acts by impairing gating. However, this behaviour is predicted if we calculate the Schild plot for the effects of calcium from model (9), with the same assumptions as for the graphs above (namely that calcium reduces efficacy by up to 50-fold when the channel is fully bound to calcium and that *E*=50 and *F*=1). This is shown in Figure 3, where the data calculated from the model are shown by the black curve and are compared with a reference line (dashed red) that has unity slope and intercept equal to the calcium microscopic dissociation constant (set to be 0.25 mM in our model, corresponding to -0.6 on the log scale).

It can be seen that the data follow a Schild-like pattern, particularly at low calcium concentration. Remarkably, the Schild plot intercept for the data is a close approximation of the true calcium dissociation constant.


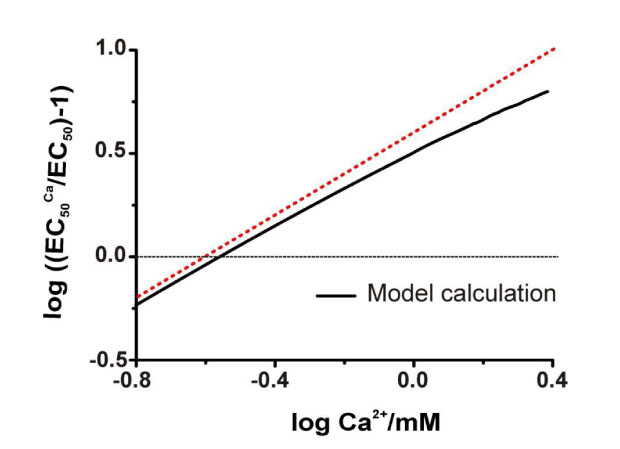


**Figure 3 – The concentration dependence of the effects of calcium is close to a Schild-like pattern, even though calcium does not act as a competitive antagonist**. The graph shows (black curve) the effect of calcium calculated from mechanism (9) and plotted as a Schild plot (see text). The dashed red line shown for reference is what a true Schild relation should look like. This is a line with unity slope that crosses the y=0 axis (dashed black horizontal line) at an intercept equal to the calcium affinity (0.25 mM e.g. -0.6 on the log scale). Mechanism (9) incorporates an intermediate state in the activation of the channel and assumes *E*=50 and *F*=1. It is also assumes that calcium binds to the resting channel with a microscopic affinity of 0.25 mM and that it reduces the value of efficacy *E* by 50-fold when bound to the channel. Note that the data calculated with these assumptions are reasonably close to the true Schild line, particularly at low calcium, where the intercept with the y=0 axis is close to the true calcium microscopic binding affinity.

*The effects of calcium and those of the competitive antagonist ACh are independent*

We can extend model (9) to include binding of the competitive antagonist ACh.

In scheme (10) , the antagonist B is modelled to bind to the resting state and to bind in a manner mutually exclusive with the binding of the agonist A, with a dissociation constant *K*_B_

(10)


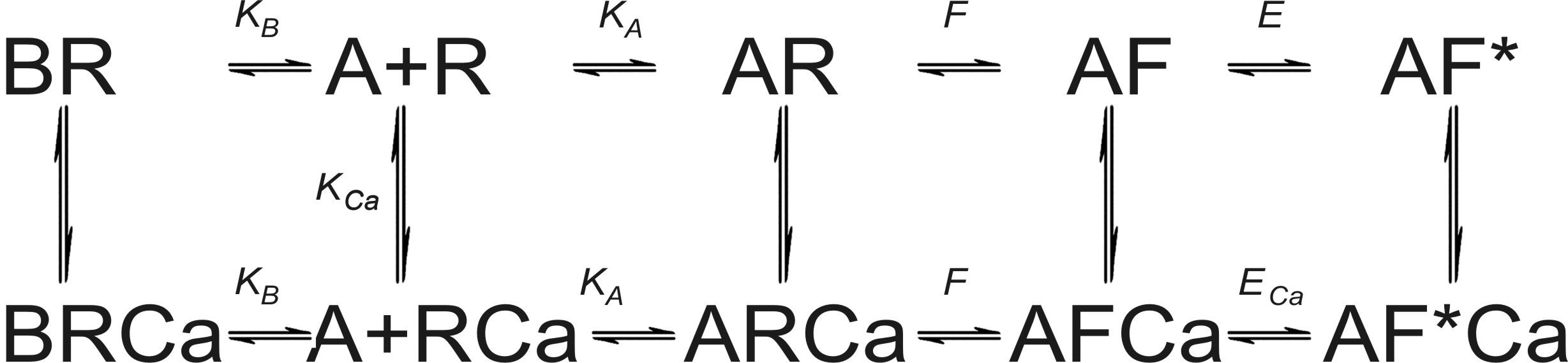


We can then calculate the dose-response curves for the agonist either in the presence of increasing concentrations of calcium (alone or in the presence of saturating ACh, e.g. 20-fold the ACh dissociation constant) or in the presence of increasing concentrations of ACh (alone or in the presence of saturating calcium, e.g. 20-fold the calcium dissociation constant). The other assumptions are the same as for mechanism (9) (namely that calcium reduces efficacy by up to 50-fold when the channel is fully bound to calcium, *E*=50 and *F*=1). It has been shown more generally [9] that in this sort of mechanism, the effects of blocker B should obey the Schild equation if the microscopic affinity of B is not changed by the other ligand (here calcium).

The graphs below (Figure 4) show that the effects of calcium and ACh are independent of each other. This is shown by the fact that calcium produces exactly the same reductions in agonist potency, seen as rightward shifts in the normalised agonist dose-response curves, whether or not high ACh concentrations are present (Figure 4A and B, respectively). The same applies to ACh (shown alone in Figure 4C and in the presence of high calcium in Figure 4D).


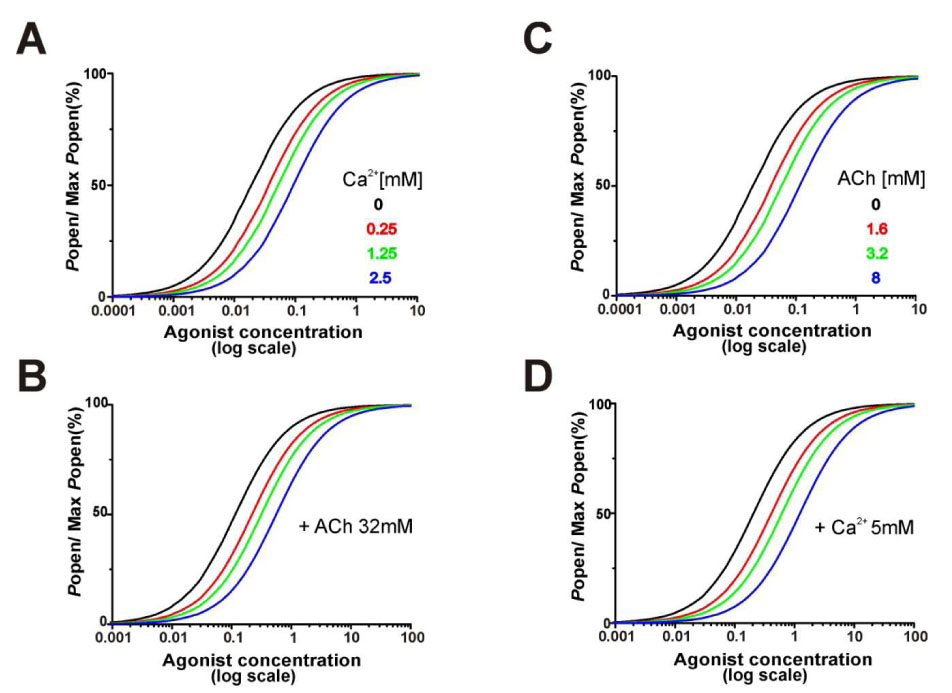


**Figure 4 – The effects of calcium and those of the competitive antagonist ACh are independent.** The graphs show agonist concentration-response curves calculated from mechanism (10). This is a mechanism that incorporates an intermediate state in the activation of the channel and assumes that the antagonist ACh can bind only to the resting state of the channel, and that ACh binding and agonist binding are mutually exclusive. The calculations are based on values of *E*=50 and *F*=1. In the model, calcium is assumed to bind to the resting state of the channel with a microscopic affinity of 0.25 mM (in line with the estimate from the Schild plot) and to reduce the value of efficacy by 50-fold when bound to the channel. ACh is assumed to bind to the resting state of the channel with a microscopic affinity of 1.6 mM (from our experimental estimate). The agonist concentration is expressed as a multiple of the (unknown) agonist microscopic dissociation constant (*K*_A_ in mechanism 10). A and B show the parallel rightward shifts in the agonist concentration-open probability curves (normalised to the maximum open probability) produced by increasing concentrations of calcium (0, 0.25,1.25 and 2.5 mM) when calcium is applied alone (A) or in the presence of a saturating concentration of ACh (32 mM, e.g. 20 times the ACh dissociation constant, B). Note that the effect of calcium is the same in A and B. C and D show the parallel rightward shifts in the agonist concentration-open probability curves (normalised to the maximum open probability) produced by increasing concentrations of ACh (0, 1.6, 3.2 and 8 mM) when ACh is applied alone (A) or in the presence of a saturating concentration of calcium (5 mM, e.g. 20 times the calcium dissociation constant, B). Note that the effect of ACh is the same in C and D.

References

1. del Castillo J, Katz B (1957) Interaction at end-plate receptors between different choline derivatives. Proc Roy Soc Lond B 146: 369-381.

2. Colquhoun D (1998) Binding, gating, affinity and efficacy: The interpretation of structure-activity relationships for agonists and of the effects of mutating receptors. Br J Pharmacol 125: 923-947.

3. Hill AV (1909) The mode of action of nicotine and curari, determined by the form of the contraction curve and the method of temperature coefficients. J Physiol 39: 361-373.

4. Maconochie DJ, Knight DE (1992) A study of the bovine adrenal chromaffin nicotinic receptor using patch clamp and concentration-jump techniques. J Physiol 454: 129-153.

5. Burzomato V, Beato M, Groot-Kormelink PJ, Colquhoun D, Sivilotti LG (2004) Single-channel behavior of heteromeric  glycine receptors: an attempt to detect a conformational change before the channel opens. J Neurosci 24: 10924-10940.

6. Mukhtasimova N, Lee WY, Wang HL, Sine SM (2009) Detection and trapping of intermediate states priming nicotinic receptor channel opening. Nature 459: 451-454.

7. Jadey S, Auerbach A (2012) An integrated catch-and-hold mechanism activates nicotinic acetylcholine receptors. J Gen Physiol 140: 17-28.

8. Schild HO (1949) pA*_x_* and competitive drug antagonism. Br J Pharmacol 4: 277-280.

9. Colquhoun D (2007) Why the Schild method is better than Schild realised. Trends Pharmacol Sci 28: 608-614.
